# Anomeric DNA Strand Displacement with α‐D Oligonucleotides as Invaders and Ethidium Bromide as Fluorescence Sensor for Duplexes with α/β‐, β/β‐ and α/α‐D Configuration

**DOI:** 10.1002/chem.202201294

**Published:** 2022-07-04

**Authors:** Aigui Zhang, Dasharath Kondhare, Peter Leonard, Frank Seela

**Affiliations:** ^1^ Laboratory of Bioorganic Chemistry and Chemical Biology Center for Nanotechnology Heisenbergstrasse 11 48149 Münster Germany; ^2^ Laboratorium für Organische und Bioorganische Chemie Institut für Chemie neuer Materialien Universität Osnabrück Barbarastrasse 7 49069 Osnabrück Germany

**Keywords:** anomeric DNA, chirality, displacement reactions, hybridization, oligonucleotides

## Abstract

DNA strand displacement is a technique to exchange one strand of a double stranded DNA by another strand (invader). It is an isothermal, enzyme free method driven by single stranded overhangs (toeholds) and is employed in DNA amplification, mismatch detection and nanotechnology. We discovered that anomeric (α/β) DNA can be used for heterochiral strand displacement. Homochiral DNA in β‐D configuration was transformed to heterochiral DNA in α‐D/β‐D configuration and further to homochiral DNA with both strands in α‐D configuration. Single stranded α‐D DNA acts as invader. Herein, new anomeric displacement systems with and without toeholds were designed. Due to their resistance against enzymatic degradation, the systems are applicable to living cells. The light‐up intercalator ethidium bromide is used as fluorescence sensor to follow the progress of displacement. Anomeric DNA displacement shows benefits over canonical DNA in view of toehold free displacement and simple detection by ethidium bromide.

## Graphical abstract

**An entirely new displacement system** based on anomeric DNA without toehold was designed and its applicability was demonstrated. Homochiral DNA in β‐D configuration was transformed to heterochiral DNA in α‐D/β‐D configuration and further to homochiral DNA with both strands in α‐D configuration. During displacement strand orientation changed from antiparallel in β‐D DNA to parallel in α/β‐D DNA and back to antiparallel in α/α‐D DNA. Reaction progress was visualized by fluorescence change using the intercalator ethidium bromide as sensor.

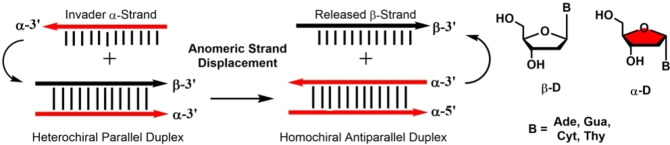

## Introduction

DNA forms a two stranded helical structure. Hydrogen bonds and vertical stacking forces hold strands together.^[1]^ Base pairing – dA pairs with dT and dG with dC – follows the Watson‐Crick principle and ensures specific recognition.^[2]^ Nevertheless, hybridization of complementary strands can lead to additional structures such as triplexes, quartets and left handed species.^[3]^ Modified nucleosides can modulate DNA structure and function.^[4]^ Particular functions of the DNA recognition system have been utilized for these applications.^[5]^ Nucleic acid chemistry, chemical biology, DNA therapeutics and diagnostics and the construction of nanomaterials make use of it.

DNA strand displacement is a method which is currently conducted to exchange one strand of a DNA by another strand.^[6]^ It represents an enzyme free copying system.^[7]^ More specific, a partially open DNA duplex is targeted by a third strand (invader). The invader strand interacts with the original duplex and releases a single strand. Then, the invader strand becomes part of the duplex. Within the whole process, three or even four strands are interacting and the reaction progresses by branch migration. The main problem of this system is the reannealing of the released strand under formation of the original duplex. This is an obstacle of evolutionary importance and the development of enzyme free replication.^[8]^

Displacement reactions are commonly performed at constant temperature. To avoid reannealing, the invader strand has to show stronger binding to the target DNA than the released strand. Most displacement protocols use nucleotide toeholds.^[9]^ Toeholds represent single‐stranded overhangs that provide additional binding capacity and are the initiation points for the strand displacement reaction. With their help, reactions are thermodynamically driven and controlled by negative Δ*G* values. Most strand displacement reactions performed so far are studied with homochiral DNA with all strands in β‐D configuration. Recently, a system was described in which DNA with all bases in D configuration was transformed to L–DNA using achiral PNA as intermediate.^[11]^ An achiral PNA‐intermediate was required as D‐DNA single strands do not hybridize with those of L–DNA.[^3b^, ^10^]

**Figure 1:**
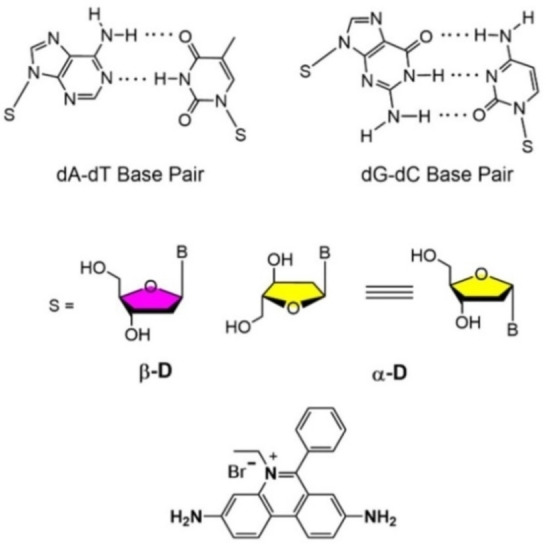
Base pairs with the sugar in β‐D or α‐D configuration and structure of ethidium bromide.

It is established that anomeric DNA forms a heterochiral recognition system.^[12]^ Duplexes with one strand in α‐D and the other in β‐D configuration are stable.^[12]^ The system was suggested by Séquin^[13]^ and the Imbach group^[12a]^ and others made important contributions to this topic.^[14]^ Also, DNA with both strands in α‐D configuration, the counterpart of canonical DNA, forms double strands.[^12d^, ^15^] They are more stable than those of canonical DNA.[^12d^, ^15^] The strand orientation of heterochiral α/β DNA is parallel,^[16]^ whereas the homochiral α/α DNA displays an antiparallel alignment[^12d^, ^15^] similar to canonical DNA. Anomeric base recognition follows the principles of Watson‐Crick base pairing with slight modifications. Recently, we have reported a series of papers on anomeric DNA modification, the effect of non‐canonical nucleobases and the function of this DNA.[^12c^, ^12d^, ^17^] The impact of silver ions on base pairing was studied.^[17b]^ In Figure 1, the nucleoside components of anomeric DNA are shown as well as the fluorescent ethidium bromide which interacts with anomeric DNAs and lights‐up upon intercalation.^[18]^

**Scheme 1:**
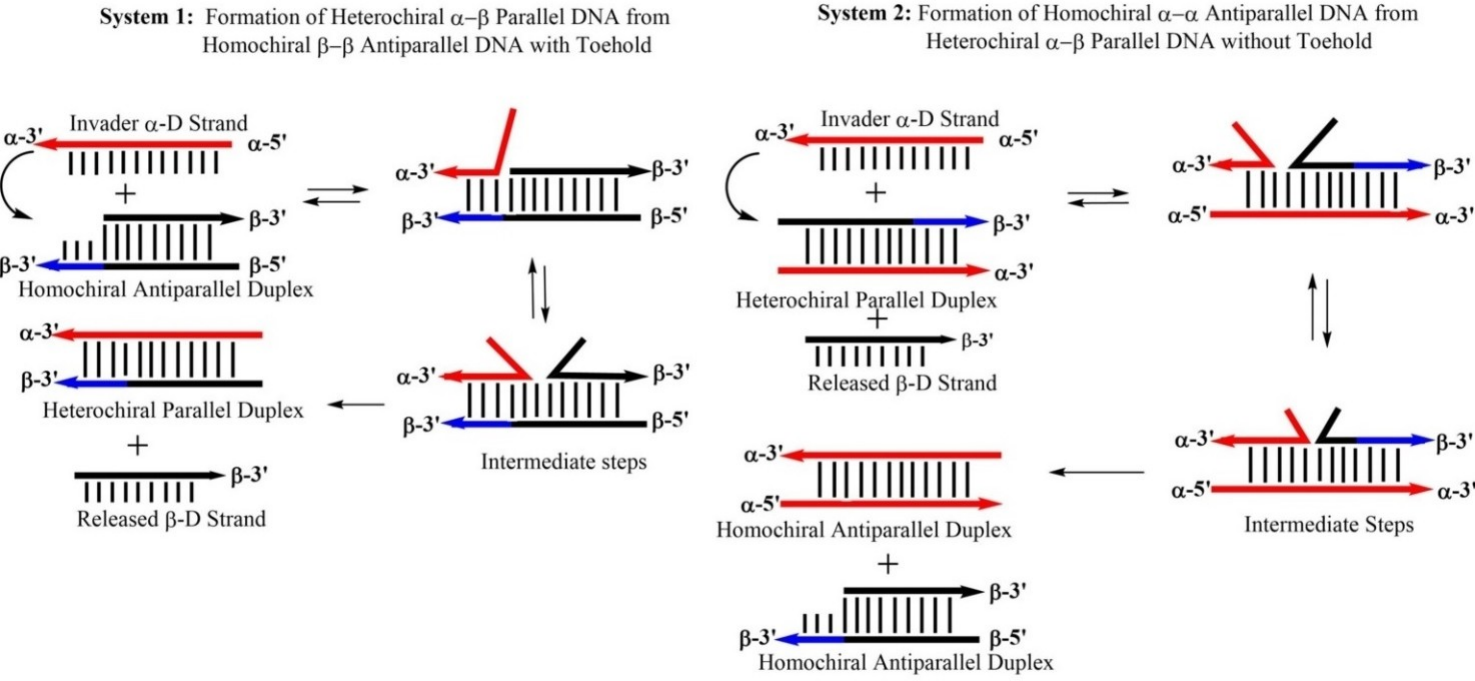
Schematic illustration of the displacement systems 1 and 2 developed in this study.

With this knowledge, we considered to construct new displacement systems on the basis of anomeric DNA. The anomeric system gives us the freedom to perform subsequent displacement reactions in the same solution. More specific, canonical DNA can be transformed in anomeric DNA with strands in α/β configuration and α/β DNA can be converted to DNA with both strands in α/α‐D configuration. Strand orientation from β/β DNA changes from antiparallel to parallel and from heterochiral DNA to homochiral α/α DNA from parallel back to antiparallel. This is an unique property of our new displacement system. To this end, several displacement systems were studied in this work. Some make use of a toehold, others work without toehold (Scheme 1). For this purpose, a new detection system was developed that enabled us to detect homochiral and heterochiral duplexes on the basis of ethidium bromide (EB) fluorescence.^[18]^

The extraordinary increase of the EB fluorescence was the basis for this design. Ethidium bromide is a standard reagent for the detection of DNA. The dye is weakly fluorescent in aqueous solution and becomes highly fluorescent, when interacting with double‐stranded DNA.^[19]^ We discovered that anomeric DNAs show characteristic fluorescence intensities. This might allow us to determine the formation of the various DNA structures formed in displacement reactions. Furthermore, the reaction progress can be detected. Ethidium bromide as a sensor represents an alternative detection system to the widely used FRET system that requires the synthesis of oligonucleotide strands modified with a terminal dye and a quencher.^[6a]^

## Results and Discussion

### Oligonucleotides used in the study

For the design of the displacement systems, a series of oligonucleotides was synthesized. Oligonucleotides are shown in Table 1. Some of them were newly prepared using solid‐phase synthesis and employing phosphoramidite chemistry; others were already described in previous papers of our laboratory.^[12d]^ For that, four α‐D and four β‐D phosphoramidites were processed. An identical synthesizer reaction cycle was employed to access canonical and anomeric oligonucleotides. Oligonucleotides were cleaved from solid support and purified by HPLC. Masses and HPLC purity profiles are shown in Table S1 and Figure S2, Supporting Information. For details see the Exp. Sect.

**Table 1:** Oligonucleotides used in this study.

Entry	Oligonucleotides
ODN‐**1**	β‐5’‐d(TAG GTC AAT ACT)
ODN‐**2**	β‐5’‐d(AGT ATT GAC CTA)
ODN‐**3**	β‐5’‐d(CAG TTA TGA)
ODN‐**4**	β‐5’‐d(ATC CAG TTA)
ODN‐**5**	β‐5’‐d(TCA TAA CTG GAT)
ODN‐**6**	β‐5’‐d(ATC CAG TTA TGA)
ODN‐**7**	α‐5‘‐d(TCA TAA CTG GAT)
ODN‐**8**	α‐5’‐d(ATC CAG TTA TGA)
ODN‐**9**	α‐5’‐d(TAG GTC AAT ACT)
ODN‐**10**	α‐5’‐d(AGT ATT GAC CTA)

### “Proof of the concept” and design of anomeric displacement systems 1 and 2

**Scheme 2:**
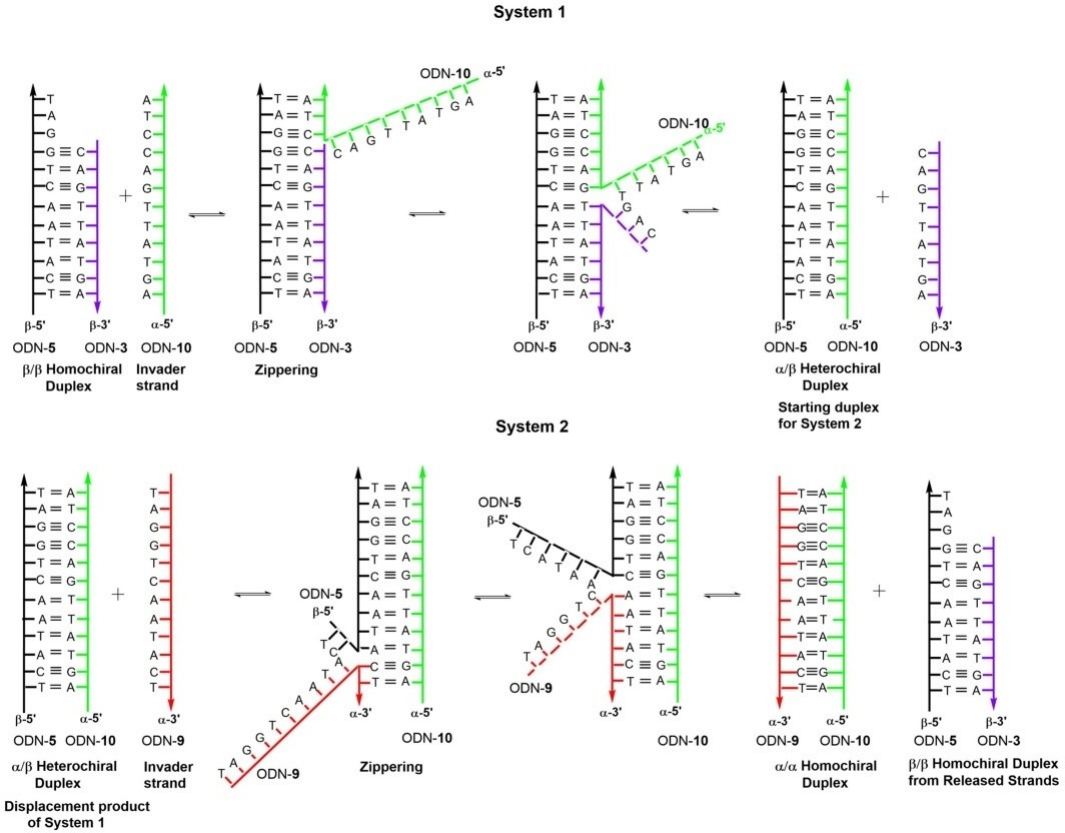
Detailed view of the possible mechanisms of the displacement reaction. Up: System 1 using a toehold‐mediated mechanism. Down: System 2 represents a displacement without using a toehold and uses the displacement product (α/β heterochiral duplex) of System 1 as starting duplex.

Commonly displacement reactions performed on DNA double helices make use of a toehold.^[9]^ Toeholds are single stranded duplex overhangs, which are not involved in base pairing. They function as initiation sites and drive the displacement reaction thermodynamically. In this study, a new type of DNA displacement reaction is designed, which uses the base recognition system of canonical DNA but shows stability differences according to the altered double helix backbone configuration. The two main systems and the input and output of the displacements are shown in Scheme 2. Whereas system 1 requires toeholds, system 2 works in the absence of toeholds with the fully matching duplexes.

In more detail, in system 1 the shorter 9‐mer β‐D strand (ODN‐**3**) of the β/β‐D duplex ODN‐**5**•ODN‐**3** is replaced by the anomeric α‐D invader strand ODN‐**10** (Scheme 2, upper part). The α‐D strand ODN‐**10** consists of the same number of base pairs as the complementary β‐D strand ODN‐**5**. It shows the same base recognition pattern as a corresponding 12‐mer β‐D strand, but hybridizes under parallel strand orientation. As the displacement reaction is thermodynamically driven, distinctions in the thermal stabilities of β/β duplexes compared to α/β duplexes might be sufficient for displacement. To investigate this matter, melting curves of β/β and α/β duplexes were measured and *T*_m_ values and thermodynamic data were determined and are summarized in Table 2 (left columns). Additionally, *T*_m_ values and thermodynamic data in the presence of ethidium bromide discussed in the next paragraph of this work are also listed Table 2 (right columns).

**Table 2:** *T*_m_ values and thermodynamic data for antiparallel and parallel stranded duplexes used in systems 1 and 2 in the absence and presence of ethidium bromide.^[a]^

	Without ethidium bromide					With ethidium bromide			
Homochiral (β/β) Duplexes (Antiparallel) without Toehold for System 1	*T*_m_^[b]^ [°C]	Δ*H*°^[c]^ [kcal mol^−1^]	Δ*S*°^[c]^ [cal/K mol]	Δ*G*°_310_^[c]^ [kcal mol^−1^]		*T*_m_^[d]^ [°C]	Δ*H*°^[c]^ [kcal mol^−1^]	Δ*S*°^[c]^ [cal/K mol]	Δ*G*°_310_^[c]^ [kcal mol^−1^]
β‐5‘‐d(TAG GTC AAT ACT) (ODN‐**1**)	47	−82	−228	−11.0		49	−85	−238	−11.5
β‐3‘‐d(ATC CAG TTA TGA) (ODN‐**2**)						(+2)			
β‐5’‐d(TCA TAA CTG GAT) (ODN‐**5**)	45	−86	−243	−10.5		47	−82	−230	−10.9
β‐3’‐d(AGT ATT GAC CTA) (ODN‐**6**)						(+2)			
Homochiral (β/β) Duplexes (Antiparallel) with Toehold for System 1									
β‐5’‐d(TCA TAA CTG GAT) (ODN‐**5**)	33	−63	−180	−7.5		36	−64	−180	−8.4
β‐3’‐d(…….ATT GAC CTA) (ODN‐**4**)						(+3)			
β‐5’‐d(TCA TAA CTG GAT) (ODN‐**5**)	31	−65	−189	−7.0		34	−66	−188	−7.7
β‐3’‐d(AGT ATT GAC…….) (ODN‐**3**)						(+3)			
Heterochiral (α/β) Duplexes (Parallel) for Systems 1 and 2									
β‐5‘‐d(TAG GTC AAT ACT) (ODN‐**1**)	45	−69	−191	−10.1		47	−75	−210	−10.4
α‐5‘‐d(ATC CAG TTA TGA) (ODN‐**8**)						(+2)			
α‐5’‐d(TCA TAA CTG GAT) (ODN‐**7**)	41	−65	−180	−9.1		43	−71	−197	−9.8
β‐5’‐d(AGT ATT GAC CTA) (ODN‐**2**)						(+2)			
α‐5‘‐d(TAG GTC AAT ACT) (ODN‐**9**)	41	−63	−175	−9.1		43	−77	−217	−9.9
β‐5‘‐d(ATC CAG TTA TGA) (ODN‐**6**)						(+2)			
β‐5’‐d(TCA TAA CTG GAT) (ODN‐**5**)	45	−73	−203	−10.2		47	−80	−223	−10.8
α‐5’‐d(AGT ATT GAC CTA) (ODN‐**10**)						(+2)			
Homochiral (α/α) Duplexes (Antiparallel) for System 2									
α‐5‘‐d(TAG GTC AAT ACT) (ODN‐**9**)	62	−124	−343	−17.5		62	−119	−329	−16.8
α‐3‘‐d(ATC CAG TTA TGA) (ODN‐**10**)						(±0)			
α‐5’‐d(TCA TAA CTG GAT) (ODN‐**7**)	62	−118	−325	−17.0		62	−122	−338	−17.3
α‐3’‐d(AGT ATT GAC CTA) (ODN‐**10**)						(±0)			

[a] Measured at 260 nm at a concentration of 5 μM+5 μM single strands at a heating rate of 1.0 °C min^−1^. 100 mM NaCl, 10 mM MgCl_2_, and 10 mM Na‐cacodylate (pH 7.0). [b] *T*_m_ values were calculated from the heating curves using the program *Meltwin 3.0*.^[20]^ [c] Data are within an error rate of ±5 %. [d] *T*_m_ values were calculated from the heating curves after adding ethidium bromide (EB) with 8.5 μM concentration using the program *Meltwin* 3.0.^[20]^

From the data listed in Table 2, it is apparent that α/β duplexes are in general slightly less stable than their β/β counterparts (Δ*T*_m_=2–6 °C). This is also confirmed by Δ*G*° values which are more favorable for the β/β duplexes (ΔΔ*G*°=0.5 to 1.9 kcal mol^−1^). So, this set‐up cannot be used as displacement system as both species of duplexes (α/β and β/β) will be present in solution. Consequently, a toehold is required to initialize the reaction. For that, the 9‐mer oligonucleotides ODN‐**3** and ODN‐**4** were prepared. Upon their hybridization with the 12‐mer ODN‐**5**, overhanging ends (toeholds) are generated either at the 3’‐ or the 5’‐end (Scheme 2). Comparison of the *T*_m_ values and thermodynamic data of the duplexes having a toehold to the full length α/β duplexes showed that the Δ*G*° values are 2.7–3.2 kcal mol^−1^ lower than for α/β duplexes. Thus, we concluded that the thermodynamic driving force should be large enough to initialize system 1.

Next, system 2 was designed and the stabilities of heterochiral duplexes with α/β configuration were compared to those with two strands in α‐D configuration (Scheme 2). This system uses the heterochiral duplex ODN‐**5**•ODN‐**10** formed in system 1 as starting material and the α‐D strand ODN‐**9** as invader. The new homochiral duplex ODN‐**9•**ODN‐**10** with both strands in α‐D configuration is then formed together with the homochiral β/β‐D duplex ODN‐**5**•ODN‐**3**. The latter is formed by the two β‐D released strands from system 1 and system 2. *T*_m_ values as well as thermodynamic data of this system shown in Table 2 reveal that Δ*G*° values are significantly more favorable for the α/α duplexes than for the corresponding α/β duplexes (ΔΔ*G*°=7.9–8.4 kcal mol^−1^). According to this, system 2 should proceed without using a toehold. In this system, the information transfer conducts from heterochiral parallel duplexes to homochiral antiparallel double helices. If both systems are combined, β/β‐D duplexes can be converted to α/α‐D double helices.

**Figure 2:**
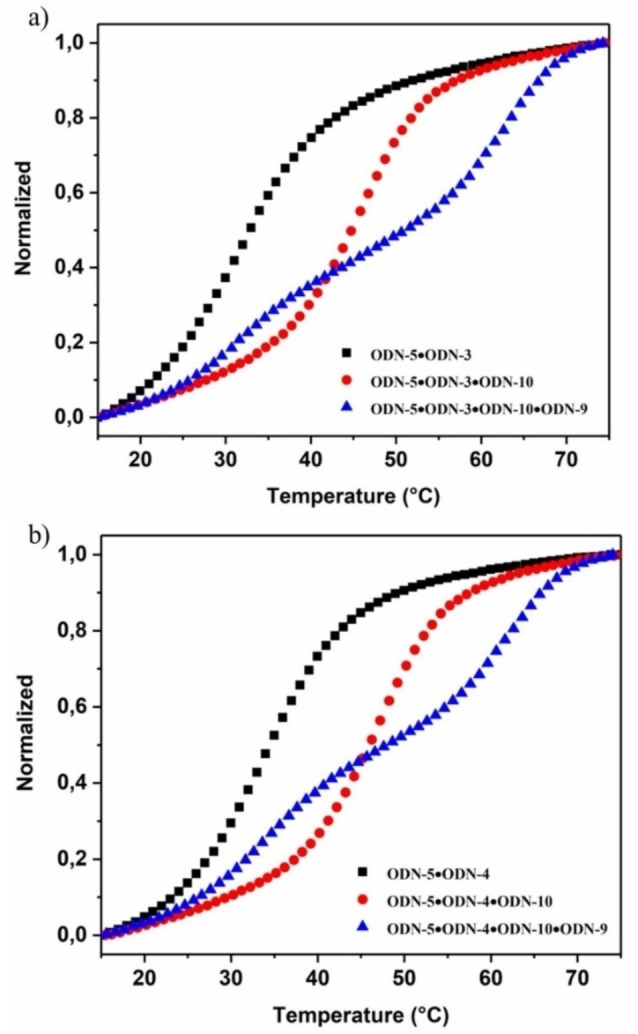
Thermal denaturation experiments of β/β antiparallel stranded duplexes before and after the addition of the α‐D invader strand and followed by addition of the second α‐D invader strand. a) ODN‐**5•**ODN‐**3** (black), ODN‐**5•**ODN‐**10** plus ODN‐**3** released (red), ODN‐**5•**ODN‐**3** and ODN‐**9•**ODN‐**10** (blue). b) ODN‐**5•**ODN‐**4** (black), ODN‐**5•**ODN‐**10** plus ODN‐**4** released (red), ODN‐**5•**ODN‐**4** and ODN‐**9•**ODN‐**10** (blue). All measurements were performed at 260 nm with 5 μM single‐strand concentration in 100 mM NaCl, 10 mM MgCl_2_, and 10 mM Na‐cacodylate (pH 7.0).

To verify this matter for the final displacement systems in the presence of invader and released strands, melting curves were measured before and after displacement for systems 1 and 2 (Figure 2). Two series of measurements were performed one with the toehold at the 3’‐end and one with toehold at the 5’‐end (Figure 2, black curves). Then, the α‐D invader strand ODN‐**10** was added to both kinds of duplexes in separate experiments and melting curves were measured again (Figure 2, red curves). After that, measurements were conducted after adding the second α‐D invader strand ODN‐**9** (Figure 2, blue curves). Sigmoidal curves were obtained in all cases. As a stability increase was observed after addition of the α‐D invader strand **10** (system 1, Figure 2), the β‐D strand is obviously released from the original duplex and the new α/β duplex was formed. This was observed for both toehold series. After addition of the second α‐D invader strand ODN‐**9** biphasic melting curves were obtained (system 2). The lower melting curves correspond to the starting β/β duplexes formed by the β‐D released strands (12‐mer and 9‐mer). The higher melting curves belong to the α/α duplex formed by the two α‐D invader strands. With respect to duplex stability, these results are in accordance to the *T*_m_ values of the ‘pure’ duplexes (Tables S2–S4, Supporting Information). ‘Pure’ means duplexes in the absence of invader or released strands. The biphasic melting profiles obtained after adding two α‐D invader strands also confirms that different species of duplexes are coexisting in solution. According to this, a combination of the anomeric displacement systems 1 and 2 is possible.

### Ethidium bromide as sensor for anomeric displacements

The studies described above used heating and cooling cycles. For the “proof of the concept”, the displacement reaction had to be performed at constant temperature without transferring energy to the system. For that, a detection system was required to follow the displacement reaction and the appearance or disappearance of species throughout the displacement reactions. The fluorescent dye ethidium bromide (EB) was selected which was initially applied for the treatment of infections by parasites^[21]^ and is now used as staining agent for the visualization of any double stranded DNA.^[22]^ The dye shows low fluorescence, that increases substantially (up to 20 fold), when it is interacting with double stranded DNA.^[23]^ Fluorescence with single strands is low. According to molecular simulation studies, EB can stack to the terminal base pair or is able to intercalate between base pairs.^[24]^ Thus, it represents a light‐up intercalator.

**Figure 3:**
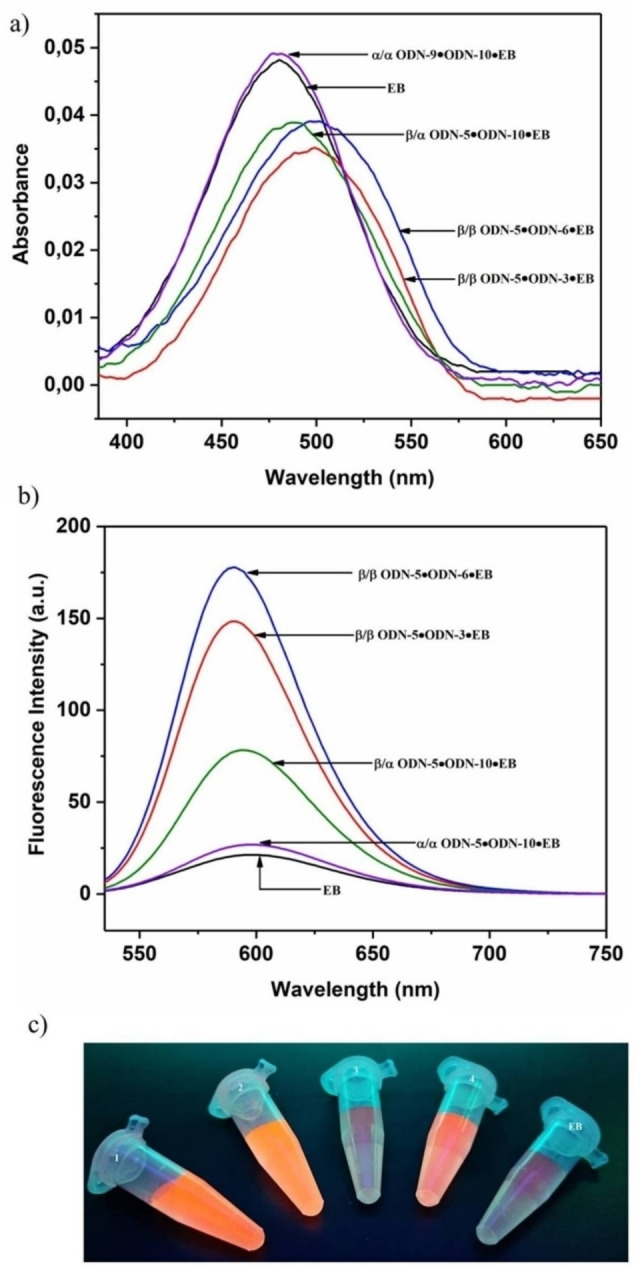
UV absorption spectra and fluorescence emission spectra for free EB and in the presence of various duplexes. a) **EB** (black), ODN‐**5•**ODN‐**3** (red), ODN‐**5•**ODN‐**6** (blue), ODN‐**5•**ODN‐**10** (green) and ODN‐**9•**ODN‐**10** (violet). b) **EB** (black), ODN‐**5•**ODN‐**3** (red), ODN‐**5•**ODN‐**6** (blue), ODN‐**5•**ODN‐**10** (green) and ODN‐**9•**ODN‐**10** (violet). c) 1) ODN‐**5•**ODN‐**3**, 2) ODN‐**5•**ODN‐**6**, 3) ODN‐**9•**ODN‐**10**, 4) ODN‐**5•**ODN‐**10**, 5) **EB**. The EB concentration was 8.5 μM and the duplex concentration was 5 μM. Measurements were performed in 100 mM NaCl, 10 mM MgCl_2_, and 10 mM Na‐cacodylate (pH 7.0).

Binding of EB to DNA can be detected by increase of fluorescence at 590 nm and excitation at 510 nm.^[21]^ Based on the specific binding characteristics of EB, we anticipated that the change from canonical to anomeric DNA can be visualized by fluorescence changes of EB. To this end, the absorption and fluorescence emission spectra of EB were measured in the absence and presence of β/β, α/β and α/α duplexes (Figure 3, Figures S8–S15, Supporting Information).

According to Figure 3, ethidium bromide shows the lowest fluorescence intensity in the absence of any DNA duplex. The highest fluorescence is observed in the presence of β/β duplexes. In this case, a shift of the UV wavelength maximum to longer wavelength is observed upon binding (∼20 nm, Figure 3a), a well‐known phenomenon described in the literature.[^23^, ^24^] For α/β duplexes, lower fluorescence emission and a slight bathochromic wavelength shift (∼5–10 nm) is observed. For α/α duplexes, the intensity is almost as low as for EB alone and no shift of the UV wavelength maximum is observed. Also, the fluorescence emission maxima are slightly shifted to shorter wavelength in case of β/β and α/β duplexes compared to EB alone, whereas no change occurs for α/α duplexes. In case of α/α duplexes also no change of the CD spectra is observed in the presence of EB (Figure S5, Supporting Information). This is contrary to β/β duplexes showing a significant increase of the CD maximum at ∼275 nm (Figure S5, Supporting Information). From these data, we anticipate that EB does not bind to α/α duplexes.

Remarkably, even the fluorescence intensity among a β/β‐D duplex with toehold and a corresponding fully‐matched 12‐mer duplex or a heterochiral α/β duplex can be distinguished. Consequently, due to the well‐defined gaps in the fluorescence intensity of EB in the presence of different DNAs, it was used to monitor the progress of the displacement reaction in the systems 1 and 2.

As it could not be excluded that EB increases duplex stability, *T*_m_ values were determined in the absence and presence of EB (Table 2, right columns). According to *T*_m_ values and thermodynamic data of Table 2, only a marginal increase of *T*_m_ values (2 °C) and slightly increased Δ*G* values (ΔΔ*G* <1 kcal mol^−1^) were observed for β/β and α/β duplexes while almost no effect for duplexes formed by two α‐D strands was detected. An impact on the thermodynamic stability of duplexes has also to be considered when the FRET detection system is used in which the dye and the quencher are usually introduced as overhanging (dangling) ends.[^3d^, ^11b^] In that regard, a significant impact on the thermodynamic stability of duplexes has been reported earlier.^[25]^ However, we anticipate that ethidium bromide binding will not obscure the progress of displacement reactions when used as sensor.

### Isothermal anomeric displacement reactions

**Figure 4:**
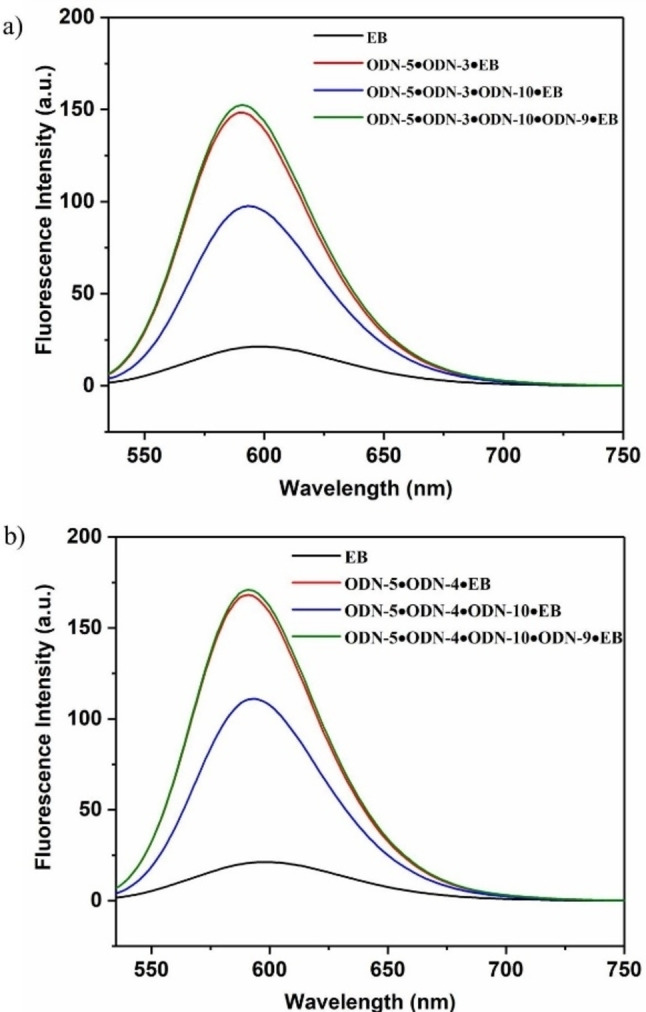
Displacement reactions of system 1 and system 2 detected by ethidium bromide as fluorescence sensor. a) Displacement reaction using a 3’‐toehold. b) Displacement reaction using a 5’‐toehold.

With the knowledge that displacements proceeded in the expected way as described above, we then investigated isothermal displacement reactions at 22 °C without transfer of heat to the systems. We started with system 1. This system consists of the antiparallel homochiral duplex ODN‐**5•**ODN‐**3** with a toehold at the 3’‐end of the duplex. Ethidium bromide was added to the duplex solution, and then the fluorescence was measured (Figure 4a, red curve). Afterwards, the α‐D invader strand ODN‐**10** was supplied and fluorescence was measured again (Figure 4a, blue curve). According to the fluorescence decrease, the 9‐mer oligonucleotide of the duplex was exchanged by the α‐anomeric 12‐mer invader ODN‐**10** under formation of the 12‐mer α/β duplex ODN‐**5•**ODN‐**10**. The outcome of system 1 was then used to establish system 2. A second displacement reaction was performed on ODN‐**5•**ODN‐**10**. For that, the 12‐mer α‐D invader strand ODN‐**9** was added. It is complementary to the α‐D invader strand ODN‐**10** used in system 1. Hence, the homochiral α/α duplex ODN‐**9•**ODN‐**10** was formed and the 12‐mer β‐D strand ODN‐**5** was released. Additionally, the two complementary released β‐D strands (9‐mer and 12‐mer) of the double displacement (system 1 followed by system 2) formed the homochiral β/β duplex ODN‐**5•**ODN‐**3**. This duplex is identical to the starting duplex of system 1 and led to a significant EB fluorescence increase.

The same reaction sequence was performed with duplex ODN‐**5•**ODN‐**4** having the overhang at the 5’‐site. The outcome of this displacement reaction was similar to that of the former experiment (Figure 4b). The fluorescence at the end of both displacement sequences was the same as at the beginning.

Unfortunately, the formation of the α/α duplex ODN‐**9•**ODN‐**10** could not directly be detected by ethidium fluorescence as the mixture is almost non‐fluorescent. However, the indirect detection of the β/β duplexes ODN‐**5•**ODN‐**3** and ODN‐**5•**ODN‐**4** confirms the formation. The displacement reactions described above demonstrate the efficiency of the new anomeric system.

**Figure 5:**
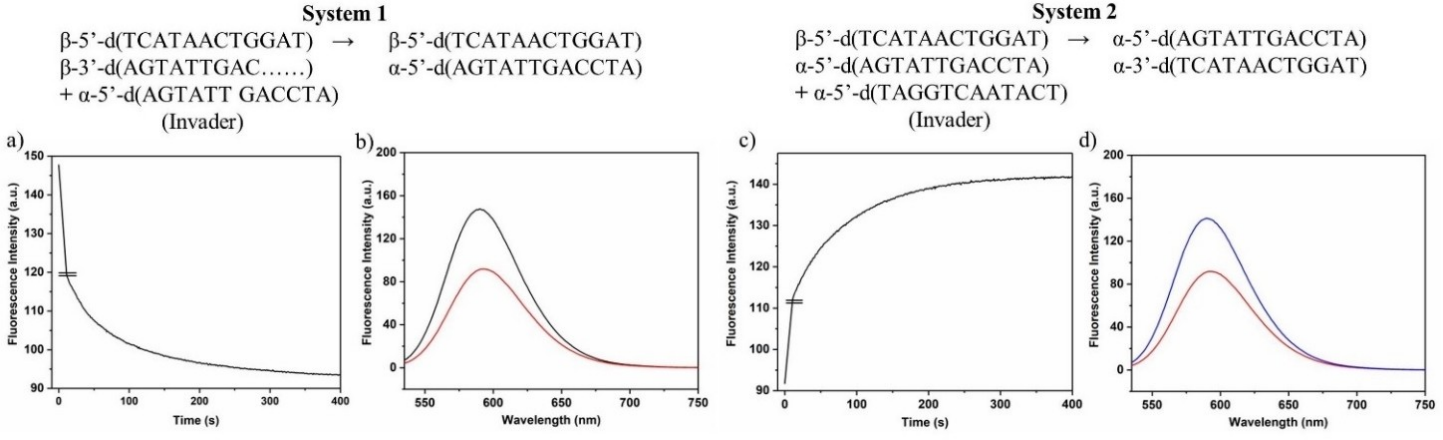
Reaction progress of displacement reactions followed by the fluorescence change of ethidium bromide (extrapolated curve). a) System 1; c) system 2. Steady‐state fluorescence emission of the starting duplex plus EB and the final duplex plus EB. b) System 1; d) system 2. All measurements were performed at 260 nm with 5 μM single‐strand concentration and 8.5 μM ethidium bromide in 100 mM NaCl, 10 mM MgCl_2_, and 10 mM Na‐cacodylate (pH 7.0).

Then, it was confirmed that ethidium bromide binding to the particular duplexes was much faster than displacement reactions. Data of Figure S1, Supporting Information show an immediate change of fluorescence after addition of EB, which was then steady within the time of the displacement reaction. Furthermore, the experiment shows that the dye was not bleaching during irradiation. Next, the progress of the displacement reactions was investigated. This was followed by time dependent recordings of fluorescence change. The graphs are shown in Figure 5 together with the steady‐state fluorescence curves of the starting duplex plus EB and the product duplex plus EB.

As the displacement reactions in both systems proceeded very fast, the original curves for the reaction progress were extrapolated to the initial value of fluorescence of the starting duplex in the presence of EB. The original curves are shown in Figure S16, Supporting Information. From the curves, it can be seen that the half‐life values for the reactions in system 1 and system 2 are below 30 s. Remarkably, the displacement reaction of system 2 in which a β‐D single strand is released from a fully matched 12‐mer α/β duplex without toehold and displaced by a 12‐mer α‐D single strand is as fast as the displacement reaction of system 1 using a toehold. Furthermore, comparison of the fluorescence emission data before and after addition of the invader strand reveals that almost quantitative displacement occurs in both systems.

As discussed above, the formation of α/α duplexes by EB fluorescence was detected indirectly by the formation of the β/β duplexes. However, the displacement reaction could be unambiguously confirmed by CD spectra.^[26]^ From previous CD studies on homochiral duplexes with both strands in α‐D configuration, it was known that their CD spectra showed a distinct negative Cotton effect around 270 nm, which is not observed for heterochiral α/β duplexes and duplexes with both strands in β‐D configuration.[^12d^, ^27^]

**Figure 6:**
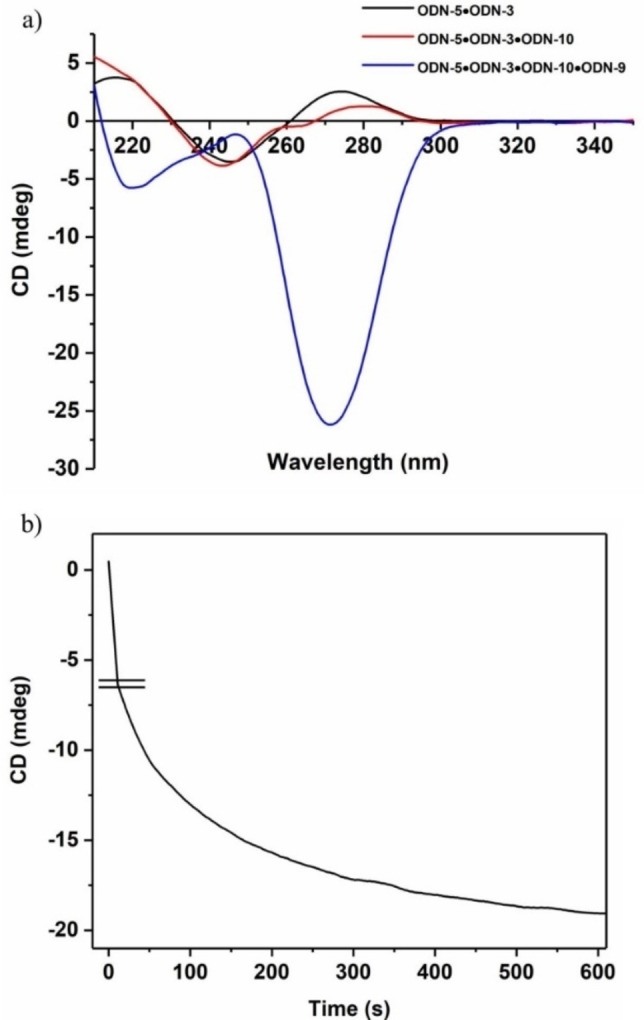
a) CD spectra of duplexes according to the displacement reactions in system 1 and system 2. Starting duplex with toehold ODN‐**5•**ODN‐**3** (black), ODN‐**5•**ODN‐**10** plus ODN‐**3** (red) (system 1), ODN‐**5•**ODN‐**3** plus ODN‐**9•**ODN‐**10** (blue) (system 2); b) Reaction progress of the displacement reaction in system 2 was measured at a fixed wavelength of 270 nm. The cell path length of the cuvette was 5 mm. All measurements were performed at 260 nm with 5 μM single‐strand concentration in 100 mM NaCl, 10 mM MgCl_2_, and 10 mM Na‐cacodylate (pH 7.0).

In the displacement reaction of system 1 followed by system 2, a smaller amplitude and a bathochromic shift of the wavelength maximum indicate α/β duplex formation (system 1), whereas a strong negative lobe at ∼270 nm unambiguously confirms α/α duplex formation (system 2) (Figure 6a). It has to be considered that in system 2 two species of duplexes are present in solution (α/α and β/β), both showing CD spectra. However, the strong amplitude of the α/α duplex is dominating.

So, the reaction progress of system 2 could be followed by a time course experiment at a fixed wavelength (270 nm) (Figure 6b). The time course clearly indicates the formation of the α/α duplex and shows that the reaction proceeds very fast. This confirms the result obtained by indirect fluorescence detection using EB. Accordingly, CD spectroscopy represents an alternative sensor system that can be used when anomeric DNA is not fluorescent.

### Anomeric displacement reactions performed with systems 3–6

Beyond that, other displacement systems based on anomeric or canonical DNA were designed. System 3 represents the most common one in the literature and is based on a toehold‐mediated displacement of one strand from a β/β‐D duplex (Figure 6). Here, a short 9‐mer strand is released and displaced by a 12‐mer β‐D strand that is complementary to the β‐D toehold strand. This system relies on canonical DNA.

**Figure 7:**
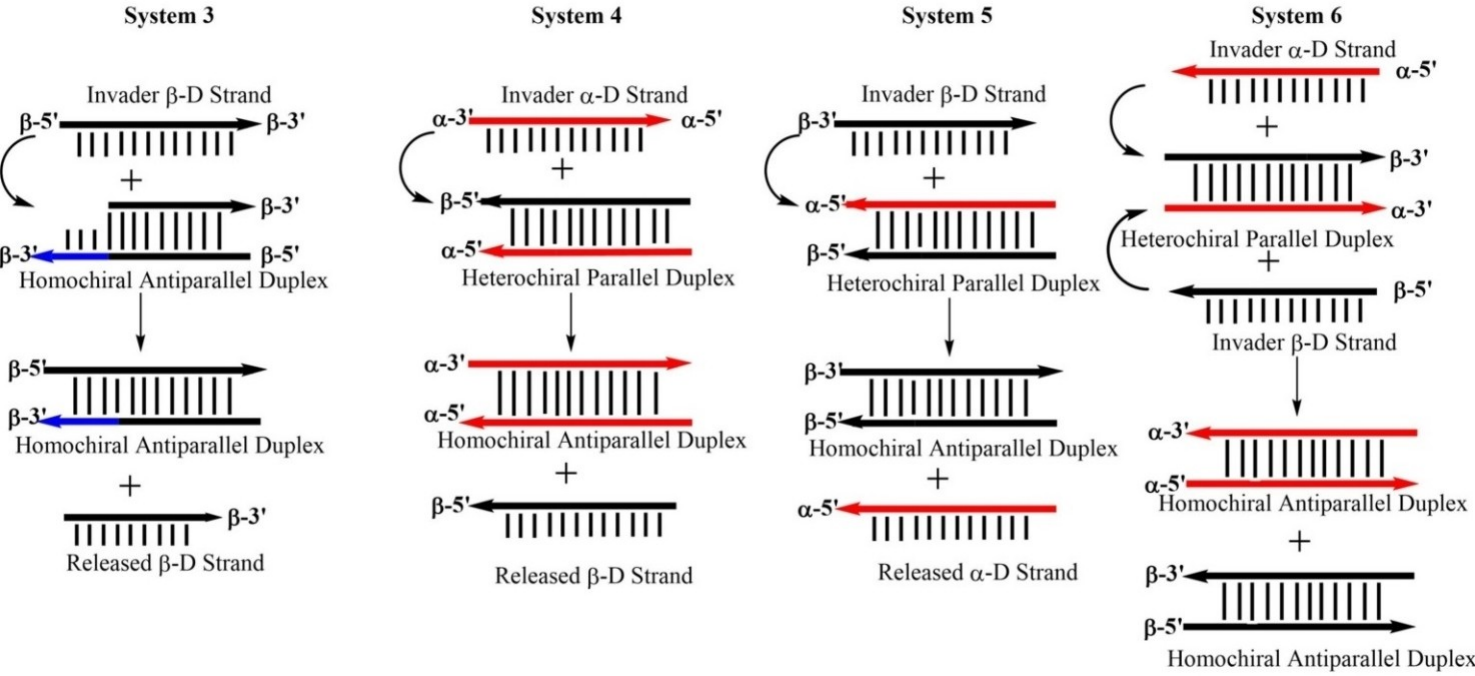
Schematic view of the displacement systems 1–6 using various combinations of anomeric double helices.

The displacement systems 4 and 5 are based on heterochiral α/β‐D double helices without toeholds as starting points. In these systems, either the α‐D strands (system 4) or the β‐D strands (system 5) of 12‐mer fully‐matched heterochiral α/β duplexes are replaced by β‐D or α‐D strands of the same base composition and recognition pattern but hybridizing in antiparallel orientation (Figure 7). In these systems, the final duplexes have α/α‐D (system 5) or β/β‐D (system 4) configuration and α‐D or β‐D released single strand are present in solution. Displacement system 6 uses a heterochiral parallel duplex as starting material. In this system, a second heterochiral parallel duplex is added formed by α‐D and β‐D single strands. These single strands are complementary to the single strands (β to β and α to α) of the starting duplex. As a consequence, thermodynamically more stable antiparallel homochiral α/α‐D and β/β‐D double helices are formed (Figure 7). In all these systems, the strand polarity is altered from parallel to antiparallel and the chirality from heterochiral to homochiral.

To evaluate the feasibility of the displacement systems 3–6, *T*_m_ measurements were performed. Melting curves are shown in Figures S3‐S4, Supporting Information and *T*_m_ values are summarized in Tables S5–S9, Supporting Information. Melting curves and *T*_m_ values indicated stability differences among the starting duplexes and the duplexes after displacement. These differences are small in system 4 (α/β→β/β) and large in system 5 (α/β→α/α). Addition of the invader duplex (system 6) results in biphasic melting with two *T*_m_ values according to formation of duplexes with β/β‐D and α/α‐D configuration.

**Figure 8:**
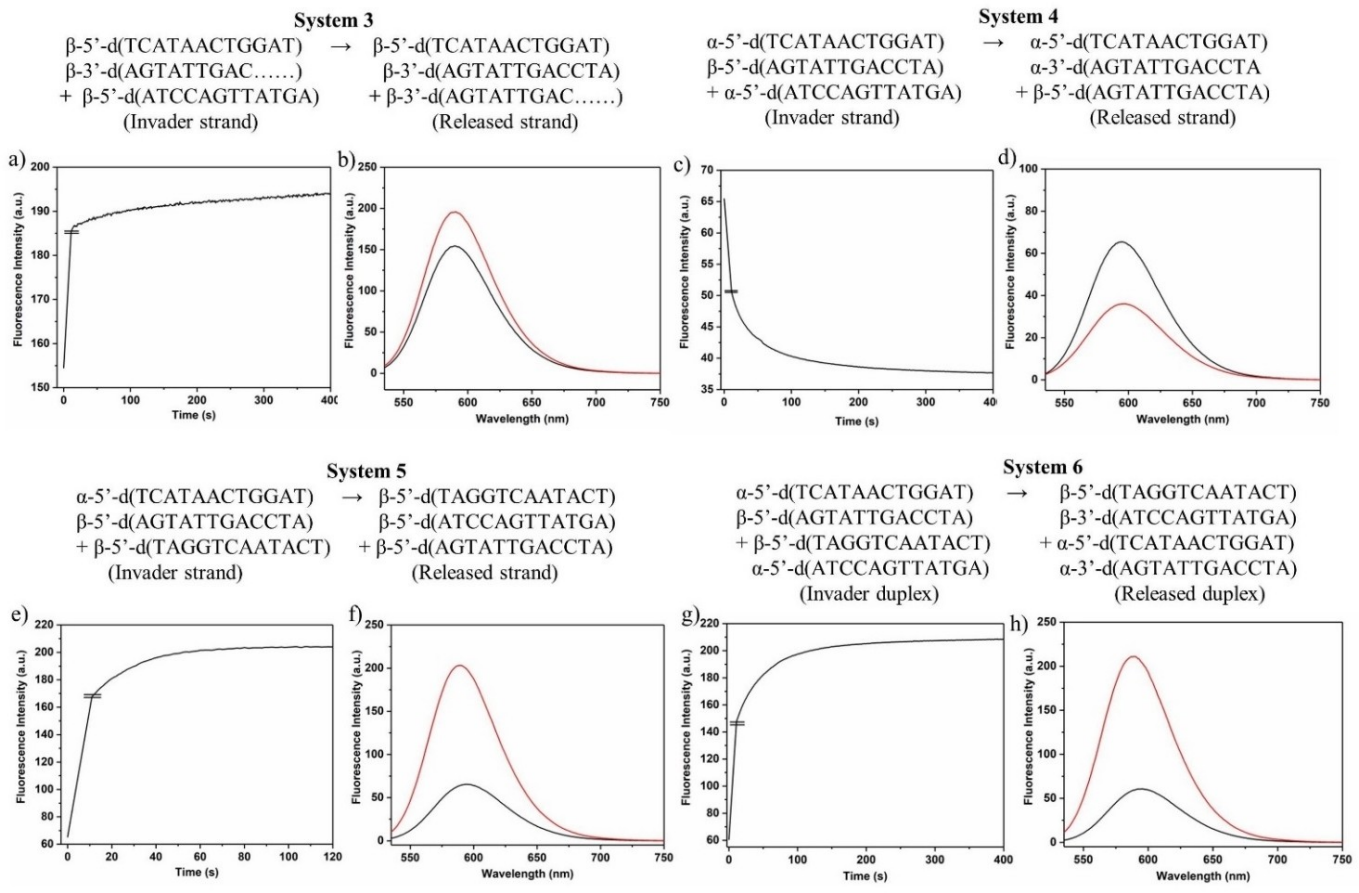
Reaction progress (extrapolated curve) of the displacement reactions according to a) system 3; c) system 4; e) system 5; f) system 6. Steady‐state fluorescence emission of the starting duplex plus EB and the final duplex plus EB. b) System 3; d) system 4; f) system 5; h) system 6. All measurements were performed at 260 nm with 5 μM single‐strand concentration and 8.5 μM EB in 100 mM NaCl, 10 mM MgCl_2_, and 10 mM Na‐cacodylate (pH 7.0).

Consequently, isothermal displacement reactions according to systems 3–6 at 22 °C could be performed and detected by fluorescence measurements using ethidium bromide as sensor at ambient temperature (Figure 8a,c,e,g). For this, fluorescence was measured before and after adding the invader strands to determine the fluorescence of EB in the presence of the starting duplex and the product duplex (Figure 8b,d,f,h). From the curves of Figure 8, it can be concluded that an almost quantitative displacement proceeds in systems 3–6. No matter, if the reaction uses a toehold or works without toehold. This is true for anomeric DNA, where heterochiral α/β duplexes are converted to β/β or α/α duplexes and also for the reaction of β/β duplexes (with toehold) to displace a β‐D 9‐mer by a β‐D 12‐mer strand (system 3). Even in system 6 where two stable α/β duplexes are brought together, the reaction is driven to the formation of α/α and β/β duplexes. The displacement in all systems could be successfully detected by using ethidium bromide. This is even valid for system 3 displacing a 9‐mer of a β/β duplex with toehold (9‐mer/12‐mer) to a fully matched 12‐mer duplex. In view of kinetics, all systems are operating very fast with half‐life values around 10 s.

**Scheme 3:**
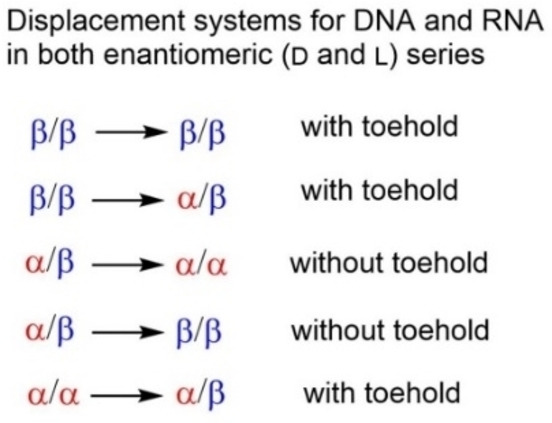
Displacement systems studied in this work and future perspectives.

A tendency is observed, that the displacement using a toehold (system 3) is faster than the displacements of systems 4–6. In summary, anomeric displacement systems 1–6 expand the utility of displacement reactions beyond existing reactions performed with canonical DNA. Scheme 3 records applications in the realm of DNA and RNA that includes enantiomeric molecules such as L–DNA or L‐RNA.

## Conclusion

Commonly, displacements are performed with homochiral DNA with all strands in β‐D configuration. Short single stranded overhangs (toeholds) are required to drive the reaction by formation of additional base pairs and for initiation of the process. In this work, an entirely new displacement system was designed and its applicability was demonstrated. This system uses anomeric DNA instead of canonical DNA. During displacement strand orientation changed from antiparallel in canonical homochiral β‐D DNA to parallel in heterochiral α/β‐D DNA and back to antiparallel in homochiral α/α‐D DNA.

For the “proof of concept”, thermodynamic stabilities of homochiral and heterochiral duplexes used in the displacement reactions were determined. Anomeric DNA displays other thermodynamic stabilities than canonical DNA. From that, the utility of the anomeric displacement system was established. According to *T*_m_ data determined from melting profiles, a toehold was required for the displacement from β/β to α/β DNA but no toehold was required when α/β DNA was converted to α/α DNA. Anomeric DNAs can coexist in solution.

Isothermal displacement reactions were performed without transferring energy to the system. To this end, a detection system was developed on the basis of the fluorescent light‐up intercalator ethidium bromide. The dye itself shows low fluorescence that increases substantially when bound to double stranded DNA. Fluorescence with single strands is low. We observed that fluorescence depends on the anomeric configuration of DNA. These changes were used to follow the progress of the anomeric displacement. Displacement reactions performed with 12‐mer duplexes took place within less than 10 s with only minor differences among the systems. The slowest displacement systems were those in which the strand orientation changed from antiparallel to parallel.

Overall, our work demonstrates that anomeric DNA can be successfully used for displacement reactions and expands the utility of this technique. The new displacement systems show properties beyond those of the systems reported for canonical DNA. According to the observations of this study, the system can be used in conjunction with RNA or with DNA or RNA in L‐enantiomeric configuration. Furthermore, DNAs formed by α/β strands or those containing two α‐strands are not cleaved by a number of nucleolytic enzymes or the cleavage is strongly reduced.^[27]^ This makes the system applicable to living systems. Ethidium bromide fluorescence enabled us to determine kinetic parameters of the displacement reactions. So, it can act as sensor for displacement reactions. As duplexes with and without toeholds can be distinguished in anomeric and canonical DNA by fluorescence changes, this light‐up sensor might be applicable to other displacement systems.

## Experimental Section

**General**: All chemicals and solvents were of laboratory grade as obtained from Acros Organics or Sigma Aldrich and were used without further purification. UV‐spectra were recorded on a Hitachi U‐3000 UV‐spectrophotometer: *λ*_max_ (ϵ) in nm, ϵ in dm^3^ mol^−1^ cm^−1^. MALDI‐TOF mass spectra of oligonucleotides were recorded on a Micro‐TOF spectrometer. The molecular masses of the oligonucleotides were determined by MALDI‐TOF mass spectrometry on a Bruker Autoflex Speed in the linear positive mode with 3‐hydroxypicolinic acid (3‐HPA) as a matrix. The thermal melting curves were measured with an Agilent Technologies Cary 100 Bio UV‐vis spectrophotometer equipped with a thermoelectrical controller. The temperature was measured continuously in the reference cell with a Pt‐100 resistor with a heating rate of 1 °C min^−1^. *T*_m_ values were determined from the melting curves using the software *Meltwin*, version 3.0.^[20]^ CD spectra were recorded on a *Jasco* J‐815 spectrometer.

**Oligonucleotide syntheses and characterization**: Solid‐phase oligonucleotide syntheses were performed with an ABI 392–08 synthesizer at 1 μmol scale (trityl‐on mode) employing the α‐D phosphoramidites^[12d]^ of the canonical bases as well as standard building blocks, giving an average coupling yield of over 95 %. After cleavage from the solid support, the oligonucleotides were deprotected in 28 % aqueous NH_3_ at 55 °C for 12 h. The 4,4’‐dimethoxytrityl containing oligonucleotides were purified by reversed‐phase HPLC (RP‐18) with a gradient system at 260 nm: (A) MeCN, (B) 0.1 m (Et_3_NH)OAc (pH 7.0)/MeCN, 95 : 5; gradient I: 0–3 min 10–15 % A in B, 3–15 min 15–50 % A in B; flow rate 0.7 mL min^−1^. The purified “trityl‐on” oligonucleotides were treated with 2.5 % CHCl_2_COOH/CH_2_Cl_2_ for 2 min at 8 °C to remove the 4,4’‐dimethoxytrityl residues. The detritylated oligomers were further purified by reversed‐phase HPLC with gradient II: 0–20 min 0–20 % A in B; 20–25 min, 20 % A in B; flow rate 0.7 mL min^−1^. The oligonucleotides were desalted on a reversed‐phase column (RP‐18) by using water for the elution of salts, and the oligonucleotides were eluted with H_2_O/MeOH (2 : 3). The oligonucleotides were lyophilized with a Speed‐Vac evaporator to yield colorless solids, which were frozen at −24 °C. The purity of all oligonucleotides was confirmed by RP‐18 HPLC (Figure S2, Supporting Information) and MALDI‐TOF mass spectrometry (Table S1, Supporting Information). The extinction coefficients ϵ_260_ (H_2_O) of the nucleosides were determined as: dA 15 400, dG 11 700, dT 8800, dC 7300, α‐dC 7300, α‐dA 15400, α‐dT 8800, α‐dG 11700 mol^‐1^ dm^3^ cm^‐1^. The extinction coefficients of the oligonucleotides were calculated from the sum of the extinction coefficients of their constituent nucleosides, with a hypochromic change of 20 % for the single strands.

### Fluorescence Studies

**General**: Fluorescence measurements were performed using a F‐2500 fluorescence or a F‐7000 fluorescence spectrophotometer (Hitachi, Tokyo, Japan). Cleaned and dried Hellma analytics cuvettes (2 mL volume) with caps were used for the fluorescence study. All measurements were performed at ambient temperature (22 °C).

**Fluorescence of oligonucleotide duplexes**: 5 μM+5 μM of single‐stranded oligonucleotides were added to 1 mL buffer (100 mM NaCl, 10 mM MgCl_2_, and 10 mM Na‐cacodylate, pH 7.0). Ethidium bromide 8.5 μM was then added to the duplex solution. The cuvette containing the above reaction mixture was shaken vigorously for uniform distribution of ethidium bromide in the solution. Then, emission spectra were recorded using excitation wavelengths determined by the fluorescence spectrophotometer.

**Reaction progress of the strand displacement reactions**: To a solution containing 5 μM of the particular duplex and 8.5 μM ethidium bromide in a fluorescence cuvette, 5 μM of the invader strand was added by a micro pipette. Then, the cuvette was shaken vigorously and set in the spectrophotometer. Then, time dependent fluorescence measurements were carried out at room temperature for a particular time interval. The instrument parameters were set as follows: Excitation wavelength (EX) 517 nm, emission wavelength (EM) 593 nm for system 1, EX 517 nm, EM 591 nm for system 2, EX 517 nm, EM 590 nm for system 3, EX 498 nm, EM 596 nm for system 4, Ex 517 nm, EM 589 nm for system 5, EX 517 nm, EM 589 nm for system 6. Slit widths were set to 10 nm for excitation and 10 nm for emission. Fluorescence data were processed using the program *OriginPro* 2017.

## Conflict of interest

The authors declare no conflict of interest.

1

## Supporting information

As a service to our authors and readers, this journal provides supporting information supplied by the authors. Such materials are peer reviewed and may be re‐organized for online delivery, but are not copy‐edited or typeset. Technical support issues arising from supporting information (other than missing files) should be addressed to the authors.

Supporting Information

## Data Availability

The data that support the findings of this study are available from the corresponding author upon reasonable request.
