# Supplementary material for: Anomeric DNA Strand Displacement with α‐D Oligonucleotides as Invaders and Ethidium Bromide as Fluorescence Sensor for Duplexes with α/β‐, β/β‐ and α/α‐D Configuration
Source: Chemistry. 2022 Jul 4;28(47):e202201294. doi: 10.1002/chem.202201294 (PMC9543212; doi:10.1002/chem.202201294)
Supplement: Supplementary file 1 — Supporting Information [file CHEM-28-0-s001.pdf]

Chemistry–A European Journal

Supporting Information

Anomeric DNA Strand Displacement with α-D Oligonucleotides as Invaders and Ethidium Bromide as Fluorescence Sensor for Duplexes with α/β-, β/β- and α/α-D Configuration

Aigui Zhang, Dasharath Kondhare, Peter Leonard, and Frank Seela*

Table of Contents

Table S1. Molecular masses of oligonucleotides determined by MALDI-TOF S2

Table S2. T_m values and thermodynamic data of parallel and antiparallel strand duplexes S2

Table S3. T_m values of parallel and antiparallel duplexes with ethidium bromide S3

Table S4. T_m values of β/β duplexes with toehold plus α-D strand S4

Table S5. T_m values of β/β duplexes with toehold plus β-D strand S5

Table S6. T_m values of α/β parallel duplexes plus β-D strand S5

Table S7. T_m values of α/β parallel duplexes plus α-D strand S6

Table S8. T_m values of α/β parallel duplexes plus β/α duplexes S7

Table S9. T_m values of displacement reactions according to systems 3-6 S8

References S8

Figure S1. Fluorescence emission of various duplexes in the presence of ethidium bromide plotted against irradiation time S9

Figure S2. HPLC elution profiles of purified oligonucleotides S9

Figure S3. Thermal denaturation curves of duplexes S10-14

Figure S4. Thermal denaturation experiments S15-16

Figure S5. CD-spectra of duplexes in presence of ethidium bromide S16-17

Figure S6. CD-spectra of α/β parallel duplexes plus β- or α-D strand S18-19

Figure S7. CD-spectra of β/β duplexes with toehold plus α-D or β-D strand S19-20

Figure S8. Fluorescence emission spectra of duplex S20-22

Figure S9. UV absorption spectra and fluorescence emission spectra of antiparallel and parallel duplexes S23

Figure S10-S15. Fluorescence emission spectra for free EB and EB bound to antiparallel and parallel duplexes S24-26

Figure S16-S17. Reaction progress of displacement reactions followed by the fluorescence change of ethidium bromide (extrapolated curve) and steady-state fluorescence emission of the starting duplex plus EB and the final duplex plus EB S26-28

Table S1. Synthesized oligonucleotides and their molecular masses determined by MALDI-TOF mass spectrometry.

Entry	Oligonucleotides	M.W. calcd.[a] exp.[b]	Entry	Oligonucleotides	M.W. calcd.[a] exp.[b])
ODN-**1**	β-5'-d(TAG GTC AAT ACT)	--	ODN-**6**	β-5'-d(ATC CAG TTA TGA)[1]	3644.4<br>3644.1
ODN-**2**	β-5'-d(AGT ATT GAC CTA)	--	ODN-**7**	α-5'-d(TCA TAA CTG GAT)[1]	3644.4<br>3644.0
ODN-**3**	β-5'-d(CAG TTA TGA)	2738.9<br>2739.3	ODN-**8**	α-5'-d(ATC CAG TTA TGA)[1]	3644.4<br>3643.5
ODN-**4**	β-5'-d(ATC CAG TTA)	2698.8<br>2698.7	ODN-**9**	α-5'-d(TAG GTC AAT ACT)[1]	3644.4<br>3644.4
ODN-**5**	β-5'-d(TCA TAA CTG GAT)[1]	3644.4<br>3644.4	ODN-**10**	α-5'-d(AGT ATT GAC CTA)[1]	3644.4<br>3644.1

[a] Calculated on the basis of the molecular mass of $[M + H]^+$. [b] Determined by MALDI-TOF mass-spectrometry as $[M + H]^+$ in the linear positive mode.

Table S2. T_m values and thermodynamic data of parallel and antiparallel stranded duplexes according to displacement systems 3-6.[a]

Homochiral Duplexes (Antiparallel)	T_m[b] [°C]	ΔH_{310} [kcal mol^{-1}]	ΔS_{310} [cal K^{-1} mol^{-1}]	ΔG_{310} [kcal mol^{-1}]
β-5'-d(TCA TAA CTG GAT) (ODN-**5**)<br>β-3'-d(AGT ATT GAC…….) (ODN-**3**)	31	-65	-188	-7.0
β-5'-d(TCA TAA CTG GAT) (ODN-**5**)<br>β-3'-d(…….ATT GAC CTA) (ODN-**4**)	33	-63	-180	-7.5
β-5'-d(TAG GTC AAT ACT) (ODN-**1**)<br>β-3'-d(ATC CAG TTA TGA) (ODN-**2**)	47	-82	-228	-11.0
β-5'-d(TCA TAA CTG GAT) (ODN-**5**)<br>β-3'-d(AGT ATT GAC TCA) (ODN-**6**)	45	-86	-243	-10.5
α-5'-d(TCA TAA CTG GAT) (ODN-**7**)<br>α-3'-d(AGT ATT GAC CTA) (ODN-**8**)	62	-122	-338	-17.3
α-5'-d(TAG GTC AAT ACT) (ODN-**9**)<br>α-3'-d(ATC CAG TTA TGA) (ODN-**10**)	62	-124	-343	-17.6
Heterochiral Duplexes (Parallel)				
β-5'-d(TAG GTC AAT ACT) (ODN-**1**)<br>α-5'-d(ATC CAG TTA TGA) (ODN-**8**)	45	-69	-191	-10.1
β-5'-d(TCA TAA CTG GAT) (ODN-**5**)<br>α-5'-d(AGT ATT GAC CTA) (ODN-**10**)	45	-73	-203	-10.2
α-5'-d(TCA TAA CTG GAT) (ODN-**7**)<br>β-5'-d(AGT ATT GAC CTA) (ODN-**2**)	41	-65	-180	-9.1
α-5'-d(TAG GTC AAT ACT) (ODN-**9**)<br>β-5'-d(ACT CAG TTA TGA) (ODN-**6**)	41	-63	-175	-9.1

[a] Measured at 260 nm at a concentration of 5 µM + 5 µM single strand with a heating rate of 1.0°C/min in 100 mM NaCl, 10 mM $MgCl_2$, and 10mM Na-cacodylate (pH 7). [b] T_m values were calculated from the heating curves using the program *Meltwin* 3.0.[2]

Table S3. T_m values of parallel and antiparallel stranded duplexes with ethidium bromide according to displacement systems 3-6.[a]

Heterochiral (α/β) Duplexes (Parallel)	T_m[b] [°C]	T_m[c] [°C] ΔT_m [°C]	Homochiral (β/β) and (α/α) Duplexes (Antiparallel)	T_m[b] [°C]	T_m[c] [°C] (ΔT_m [°C]
α-5'-d(TCA TAA CTG GAT) (ODN-**7**)<br>β-5'-d(AGT ATT GAC CTA) (ODN-**2**)	41	43 (+2)	β-5'-d(TAG GTC AAT ACT) (ODN-**1**)<br>β-3'-d(ATC CAG TTA TGA) (ODN-**2**)	47	49 (+2)
α-5'-d(TAG GTC AAT ACT) (ODN-**9**)<br>β-5'-d(ACT CAG TTA TGA) (ODN-**6**)	41	43 (+2)	β-5'-d(TCA TAA CTG GAT) (ODN-**5**)<br>β-3'-d(AGT ATT GAC CTA) (ODN-**6**)	45	47 (+2)
β-5'-d(TAG GTC AAT ACT) (ODN-**1**)<br>α-5'-d(ATC CAG TTA TGA) (ODN-**8**)	45	47 (+2)	α-5'-d(TCA TAA CTG GAT) (ODN-**7**)<br>α-3'-d(AGT ATT GAC CTA) (ODN-**8**)	62	62 (0)
β-5'-d(TCA TAA CTG GAT) (ODN-**5**)<br>α-5'-d(AGT ATT GAC CTA)(ODN-**10**)	45	47 (+2)	α-5'-d(TAG GTC AAT ACT) (ODN-**9**)<br>α-3'-d(ATC CAG TTATGA)(ODN-**10**)	62	62 (0)
			β-5'-d(TCA TAA CTG GAT) (ODN-**5**)<br>β-3'-d(AGT ATT GAC…….) (ODN-**3**)	31	34 (+3)
			β-5'-d(TCA TAA CTG GAT) (ODN-**5**)<br>β-3'-d(…….ATT GAC CTA) (ODN-**4**)	33	37 (+4)

[a] Measured at 260 nm at a concentration of 5 µM + 5 µM single strand with a heating rate of 1.0°C/min in 100 mM NaCl, 10 mM $MgCl_2$, and 10 mM Na-cacodylate (pH 7.0). [b] T_m values were calculated from the heating curves using the program *Meltwin 3.0*. [c] T_m values were calculated from the heating curves after adding Ethidium bromide (EB) with 8.5 µM concentration.

Table S4. T_m values of β/β duplexes with toehold plus corresponding α-D invader strands according to displacement systems 1 and 2.[a]

β/β Duplexes<br>(Antiparallel)	T_m[b]<br>[°C]	α/β Duplexes System 1<br>(Parallel)	T_m[c]<br>[°C]	β/β and α/α Duplexes System 2<br>(Antiparallel)	T_m[d]<br>[°C]
**Original Strand with Toehold**<br>β-5’-d(TCA TAA CTG GAT) (ODN-**5**)<br>β-3’-d(AGT ATT GAC…….) (ODN-**3**)<br><br>**Invader Input**<br>α-5’-d(AGT ATT GAC CTA) (ODN-**10**)	31	β-5’-d(TCA TAA CTG GAT) (ODN-**5**)<br>α-5’-d(AGT ATT GAC CTA) (ODN-**10**)<br>+<br>β-3’-d(AGT ATT GAC) (ODN-**3**)<br><br>**Invader Input**<br>α-3’-d(ATC CAG TTA TGA) (ODN-**10**)	46	α-5’-d(TAG GTC AAT ACT) (ODN-**9**)<br>α-3’-d(ATC CAG TTA TGA) (ODN-**10**)<br>+<br>β-5’-d(TCA TAA CTG GAT) (ODN-**5**)<br>β-3’-d(AGT ATT GAC) (ODN-**3**)	63<br><br>30
**Original Strand with Toehold**<br>β-5’-d(TCA TAA CTG GAT) (ODN-**5**)<br>β-3’-d(…….ATT GAC CTA) (ODN-**4**)<br><br>**Invader Input**<br>α-5’-d(AGT ATT GAC CTA) (ODN-**10**)	33	β-5’-d(TCA TAA CTG GAT) (ODN-**5**)<br>α-5’-d(AGT ATT GAC CTA) (ODN-**10**)<br>+<br>β-3’-d(ATT GAC CTA) (ODN-**4**)<br><br>**Invader Input**<br>α-3’-d(ATC CAG TTA TGA) (ODN-**10**)<br>+	46	α-5’-d(TAG GTC AAT ACT) (ODN-**9**)<br>α-3’-d(ATC CAG TTA TGA) (ODN-**10**)<br>+<br>β-5’-d(TCA TAA CTG GAT) (ODN-**5**)<br>β-3’-d(ATT GAC CTA……..) (ODN-**4**)	63<br><br>32
**Pure Anomeric Duplexes**					
β-5’-d(TCA TAA CTG GAT) (ODN-**5**)<br>α-5’-d(AGT ATT GAC CTA) (ODN-**10**)	45				
α-5’-d(TAG GTC AAT ACT) (ODN-**9**)<br>α-3’-d(ATC CAG TTA TGA) (ODN-**10**)	62				

[a] Measured at 260 nm at a concentration of 5 µM + 5 µM single strand with a heating rate of 1.0°C/min in 100 mM NaCl, 10 mM $MgCl_2$, and 10 mM Na-cacodylate (pH 7.0). [b] T_m values were calculated from the heating curves using the program *Meltwin 3.0*.[2] [c] T_m values were calculated from the heating curves after adding the α-D invader strand ODN-**10** with 5 µM concentration. [d] T_m values were calculated from the heating curves after adding the second α-D invader strand ODN-**9** with 5 µM concentration.

Table S5. T_m values of β/β duplexes with toehold plus β-D invader strand.[a]

β/β Duplexes with Toehold<br>(Antiparallel)	T_m[b]<br>[°C]	Homochiral (β/β) Duplexes<br>(Antiparallel)	T_m[c]<br>[°C]
**Starting Duplex with Toehold**<br>β-5'-d(TCA TAA CTG GAT) (ODN-**5**)<br>β-3'-d(AGT ATT GAC…….) (ODN-**3**)<br>+<br>**Invader Strand**<br>β-3'-d(AGT ATT GAC CTA) (ODN-**6**)	31	**New β/β Duplex (First Displacement)**<br>β-5'-d(TCA TAA CTG GAT) (ODN-**5**)<br>β-3'-d(AGT ATT GAC CTA) (ODN-**6**)<br>+<br>**Released Strand**<br>β-3'-d(AGT ATT GAC) (ODN-**3**)	46
**Starting Duplex with Toehold**<br>β-5'-d(TCA TAA CTG GAT) (ODN-**5**)<br>β-3'-d(…….ATT GAC CTA) (ODN-**4**)<br>+<br>**Invader Strand**<br>β-3'-d(AGT ATT GAC CTA) (ODN-**6**)	33	**New β/β Duplex**<br>β-5'-d(TCA TAA CTG GAT) (ODN-**5**)<br>β-3'-d(AGT ATT GAC CTA) (ODN-**6**)<br>+<br>**Released Strand**<br>β-3'-d(ATT GAC CTA) (ODN-**4**)	46
**Pure Duplex without Released Strand**			
β-5'-d(TCA TAA CTG GAT) (ODN-**5**)<br>β-3'-d(AGT ATT GAC CTA) (ODN-**6**)	45		

[a] Measured at 260 nm at a concentration of 5 µM + 5 µM single strand at a heating rate of 1.0°C/min in 100 mM NaCl, 10 mM $MgCl_2$, and 10 mM Na-cacodylate (pH 7.0). [b] T_m values were calculated from the heating curves using the program *Meltwin 3.0*.[2] [c] T_m values were calculated from the heating curves after adding the α-D invader strand with 5 µM concentration.

Table S6. T_m values of α/β duplexes plus β-D invader strand.[a]

Heterochiral (α/β) Duplexes<br>(Parallel)	T_m[b]<br>[°C]	Homochiral (β/β) Duplexes<br>(Antiparallel)	T_m[c]<br>[°C]
**Original Strand**<br>α-5'-d(TCA TAA CTG GAT) (ODN-**7**)<br>β-5'-d(AGT ATT GAC CTA) (ODN-**2**)<br>**Invader Input**<br>β-5'-d(TAG GTC AAT ACT) (ODN-**1**)	41	β-5'-d(TAG GTC AAT ACT) (ODN-**1**)<br>β-3'-d(ATC CAG TTA TGA) (ODN-**2**)<br>+<br>α-5'-d(TCA TAA CTG GAT) (ODN-**7**)	47
**Original Strand**<br>α-5'-d(TAG GTC AAT ACT) (ODN-**9**)<br>β-5'-d(ATC CAG TTA TGA) (ODN-**6**)<br>**Invader Input**<br>β-5'-d(TCA TAA CTG GAT) (ODN-**5**)	41	β-5'-d(TCA TAA CTG GAT) (ODN-**5**)<br>β-3'-d(AGT ATT GAC CTA) (ODN-**6**)<br>+<br>α-5'-d(TAG GTC AAT ACT) (ODN-**9**)	46
**Original Strand**<br>β-5'-d(TAG GTC AAT ACT) (ODN-**1**)<br>α-5'-d(ATC CAG TTA TGA) (ODN-**8**)<br>**Invader Input**<br>β-3'-d(ATC CAG TTA TGA) (ODN-**2**)	45	β-5'-d(TAG GTC AAT ACT) (ODN-**1**)<br>β-3'-d(ATC CAG TTA TGA) (ODN-**2**)<br>+<br>α-5'-d(TCA TAA CTG GAT) (ODN-**8**)	49
**Original Strand**<br>β-5'-d(TCA TAA CTG GAT) (ODN-**5**)<br>α-5'-d(AGT ATT GAC CTA) (ODN-**10**)<br>**Invader Input**<br>β-3'-d(AGT ATT GAC TCA) (ODN-**6**)	45	β-5'-d(TCA TAA CTG GAT) (ODN-**5**)<br>β-3'-d(AGT ATT GAC TCA) (ODN-**6**)<br>+<br>α-5'-d(AGT ATT GAC CTA) (ODN-**10**)	47

[a] Measured at 260 nm at a concentration of 5 µM + 5 µM single strand with a heating rate of 1.0°C/min in 100 mM NaCl, 10 mM $MgCl_2$, and 10 mM Na-cacodylate (pH 7.0). [b] T_m values were calculated from the heating curves using the program *Meltwin 3.0*.[2] [c] T_m values were calculated from the heating curves after adding the β-D invader strand with 5 µM concentration.

Table S7. T_m values of α/β duplexes plus corresponding α-D invader strand.[a]

Heterochiral (α/β) Duplexes (Parallel)	T_m[b] [°C]	Homochiral (α/α) Duplexes (Antiparallel)	T_m[c] [°C]
**Original Strand**<br>α-5'-d(TCA TAA CTG GAT) (ODN-**7**)<br>β-5'-d(AGT ATT GAC CTA) (ODN-**2**)<br><br>**Invader Input**<br>α-3'-d(AGT ATT GAC CTA) (ODN-**8**)	41	α-5'-d(TCA TAA CTG GAT) (ODN-**7**)<br>α-3'-d(AGT ATT GAC CTA) (ODN-**8**)<br>+<br>β-5'-d(AGT ATT GAC CTA) (ODN-**2**)	62
**Original Strand**<br>α-5'-d(TAG GTC AAT ACT) (ODN-**9**)<br>β-5'-d(ATC CAG TTA TGA) (ODN-**6**)<br><br>**Invader Input**<br>α-3'-d(ATC CAG TTA TGA) (ODN-**10**)	41	α-5'-d(TCA TAA CTG GAT) (ODN-**9**)<br>α-3'-d(ATC CAG TTA TGA) (ODN-**10**)<br>+<br>β-5'-d(ACT CAG TTA TGA) (ODN-**6**)	62
**Original Strand**<br>β-5'-d(TAG GTC AAT ACT) (ODN-**1**)<br>α-5'-d(ATC CAG TTA TGA) (ODN-**8**)<br><br>**Invader Input**<br>α-5'-d(TCA TAA CTG GAT) (ODN-**7**)	45	α-5'-d(TCA TAA CTG GAT) (ODN-**7**)<br>α-3'-d(AGT ATT GAC CTA) (ODN-**8**)<br>+<br>β-5'-d(TAG GTC AAT ACT) (ODN-**1**)	62
**Original Strand**<br>β-5'-d(TCA TAA CTG GAT) (ODN-**5**)<br>α-5'-d(AGT ATT GAC CTA) (ODN-**10**)<br><br>**Invader Input**<br>α-5'-d(TAG GTC AAT ACT) (ODN-**9**)	45	α-5'-d(TAG GTC AAT ACT) (ODN-**9**)<br>α-3'-d(ATC CAG TTA TGA) (ODN-**10**)<br>+<br>β-5'-d(TCA TAA CTG GAT) (ODN-**5**)	62

[a] Measured at 260 nm at a concentration of 5 µM + 5 µM single strand at a heating rate of 1.0°C/min in 100 mM NaCl, 10 mM $MgCl_2$, and 10 mM Na-cacodylate (pH 7.0). [b] T_m values were calculated from the heating curves using the program *Meltwin 3.0*.[2] [c] T_m values were calculated from the heating curves after adding the α-D invader strand with 5 µM concentration.

Table S8. T_m values of α/β duplexes plus corresponding β/α invader duplexes.[a]

<table>
<tr><th>Heterochiral (α/β) Duplexes
(Parallel)</th><th>T_m[b]
[°C]</th><th>Homochiral (β/β and α/α) Duplexes
(Antiparallel)</th><th>T_m[c]
[°C]</th></tr>
<tr><td>Original Strand
α-5’-d(TCA TAA CTG GAT) (ODN-7)
β-5’-d(AGT ATT GAC CTA) (ODN-2)

Invader Input
β-5’-d(TAG GTC AAT ACT) (ODN-1)
α-5’-d(ATC CAG TTA TGA) (ODN-8)</td><td>41</td><td>β-5’-d(TAG GTC AAT ACT) (ODN-1)
β-3’-d(ATC CAG TTA TGA) (ODN-2)
+
α-5’-d(TCA TAA CTG GAT) (ODN-7)
α-3’-d(AGT ATT GAC CTA) (ODN-8)</td><td>47

60</td></tr>
<tr><td>Original Strand
α-5’-d(TAG GTC AAT ACT) (ODN-9)
β-5’-d(ATC CAG TTA TGA) (ODN-6)

Invader Input
β-5’-d(TCA TAA CTG GAT) (ODN-5)
α-5’-d(AGT ATT GAC CTA) (ODN-10)</td><td>41</td><td>β-5’-d(TCA TAA CTG GAT) (ODN-5)
β-3’-d(AGT ATT GAC TCA) (ODN-6)
+
α-5’-d(TAG GTC AAT ACT) (ODN-9)
α-3’-d(ATC CAG TTA TGA) (ODN-10)</td><td>45

59</td></tr>
<tr><td>Original Strand
β-5’-d(TAG GTC AAT ACT) (ODN-1)
α-5’-d(ATC CAG TTA TGA) (ODN-8)

Invader Input
α-5’-d(TCA TAA CTG GAT) (ODN-7)
β-5’-d(AGT ATT GAC CTA) (ODN-2)</td><td>45</td><td>β-5’-d(TAG GTC AAT ACT) (ODN-1)
β-3’-d(ATC CAG TTA TGA) (ODN-2)
+
α-5’-d(TCA TAA CTG GAT) (ODN-7)
α-3’-d(AGT ATT GAC CTA) (ODN-8)</td><td>46

59</td></tr>
<tr><td>Original Strand
β-5’-d(TCA TAA CTG GAT) (ODN-5)
α-5’-d(AGT ATT GAC CTA) (ODN-10)

Invader Input
α-5’-d(TAG GTC AAT ACT) (ODN-9)
β-5’-d(ATC CAG TTA TGA) (ODN-6)</td><td>45</td><td>β-5’-d(TCA TAA CTG GAT) (ODN-5)
β-3’-d(AGT ATT GAC TCA) (ODN-6)
+
α-5’-d(TAG GTC AAT ACT) (ODN-9)
α-3’-d(ATC CAG TTA TGA) (ODN-10)</td><td>44

59</td></tr>
</table>

[a] Measured at 260 nm at a concentration of 5 µM + 5 µM single strand with a heating rate of 1.0°C/min in 100 mM NaCl, 10 mM $MgCl_2$, and 10 mM Na-cacodylate (pH 7.0). [b] T_m values were calculated from the heating curves using the program *Meltwin 3.0*.[2] [c] T_m values were calculated from the heating curves after adding the corresponding α/β invader duplex with 5 µM concentration.

Table S9. T_m values of displacement reactions according to systems 3-6.[a]

Homochiral (β/β) Duplexes<br>with toehold<br>(System 3)	T_m [b]<br>[°C]	Homochiral (β/β) Duplexes	T_m [b]<br>[°C]
β-5’-d(TCA TAA CTG GAT) (ODN-**5**)<br>β-3’-d(AGT ATT GAC…….) (ODN-**3**)<br>+<br>β-3’-d(AGT ATT GAC CTA) (ODN-**6**)	31	β-5’-d(TCA TAA CTG GAT) (ODN-**5**)<br>β-3’-d(AGT ATT GAC CTA) (ODN-**6**)<br>+<br>β-5’-d(AGT ATT GAC…….) (ODN-**3**)	46
β-5’-d(TCA TAA CTG GAT) (ODN-**5**)<br>β-3’-d(…….ATT GAC CTA) (ODN-**4**)<br>+<br>β-3’-d(AGT ATT GAC CTA) (ODN-**6**)	33	β-5’-d(TCA TAA CTG GAT) (ODN-**5**)<br>β-3’-d(AGT ATT GAC CTA) (ODN-**6**)<br>+<br>β-3’-d(…….ATT GAC CTA) (ODN-**4**)	46
Heterochiral (α/β) Duplexes<br>(System 4)		Homochiral (β/β) Duplexes	
α-5’-d(TCA TAA CTG GAT) (ODN-**7**)<br>β-5’-d(AGT ATT GAC CTA) (ODN-**2**)<br>+<br>β-5’-d(TAG GTC AAT ACT) (ODN-**1**)	41	β-5’-d(TAG GTC AAT ACT) (ODN-**1**)<br>β-3’-d(ATC CAG TTA TGA) (ODN-**2**)<br>+<br>α-5’-d(TCA TAA CTG GAT) (ODN-**7**)	47
α-5’-d(TAG GTC AAT ACT) (ODN-**9**)<br>β-5’-d(ATC CAG TTA TGA) (ODN-**6**)<br>+<br>β-5’-d(TCA TAA CTG GAT) (ODN-**5**)	41	β-5’-d(TCA TAA CTG GAT) (ODN-**5**)<br>β-3’-d(AGT ATT GAC CTA) (ODN-**6**)<br>+<br>α-5’-d(TAG GTC AAT ACT) (ODN-**9**)	46
Heterochiral (α/β) Duplexes<br>(System 5)		Homochiral (α/α) Duplexes	
α-5’-d(TCA TAA CTG GAT) (ODN-**7**)<br>β-5’-d(AGT ATT GAC CTA) (ODN-**2**)<br>+<br>α-3’-d(AGT ATT GAC CTA) (ODN-**8**)	41	α-5’-d(TCA TAA CTG GAT) (ODN-**7**)<br>α-3’-d(AGT ATT GAC CTA) (ODN-**8**)<br>+<br>β-5’-d(AGT ATT GAC CTA) (ODN-**2**)	62
α-5’-d(TAG GTC AAT ACT) (ODN-**9**)<br>β-5’-d(ACT CAG TTA TGA) (ODN-**6**)<br>+<br>α-5’-d(TCA TAA CTG GAT) (ODN-**9**)	41	α-5’-d(TCA TAA CTG GAT) (ODN-**9**)<br>α-3’-d(ATC CAG TTA TGA) (ODN-**10**)<br>+<br>β-5’-d(ACT CAG TTA TGA) (ODN-**6**)	62
Heterochiral (α/β) Duplexes<br>(System 6)		Homochiral (β/β and α/α) Duplexes	
α-5’-d(TCA TAA CTG GAT) (ODN-**7**)<br>β-5’-d(AGT ATT GAC CTA) (ODN-**2**)<br>+<br>β-5’-d(TAG GTC AAT ACT) (ODN-**1**)<br>α-3’-d(AGT ATT GAC CTA) (ODN-**8**)	41	β-5’-d(TAG GTC AAT ACT) (ODN-**1**)<br>β-3’-d(ATC CAG TTA TGA) (ODN-**2**)<br>+<br>α-5’-d(TCA TAA CTG GAT) (ODN-**7**)<br>α-3’-d(AGT ATT GAC CTA) (ODN-**8**)	47<br><br>60
α-5’-d(TAG GTC AAT ACT) (ODN-**9**)<br>β-5’-d(ACT CAG TTA TGA) (ODN-**6**)<br>+<br>α-3’-d(ATC CAG TTA TGA) (ODN-**10**)<br>β-5’-d(TCA TAA CTG GAT) (ODN-**5**)	41	β-5’-d(TCA TAA CTG GAT) (ODN-**5**)<br>β-3’-d(AGT ATT GAC TCA) (ODN-**6**)<br>+<br>α-5’-d(TAG GTC AAT ACT) (ODN-**9**)<br>α-3’-d(ATC CAG TTA TGA) (ODN-**10**)	45<br><br>59

[a] Measured at 260 nm at a concentration of 5 µM + 5 µM single strand at a heating rate of 1.0°C/min in 100 mM NaCl, 10 mM $MgCl_2$, and 10 mM Na-cacodylate (pH 7.0). [b] T_m values were calculated from the heating curves using the program *Meltwin 3.0*.[2] The invader strands were added with 5 µM concentration.

References

[1] A. Zhang, P. Leonard, F. Seela, *Chem. Eur. J.* **2022**, *28*, e202103872.

[2] J. A. McDowell, D. H. Turner, *Biochemistry* **1996**, *35*, 14077-14089.

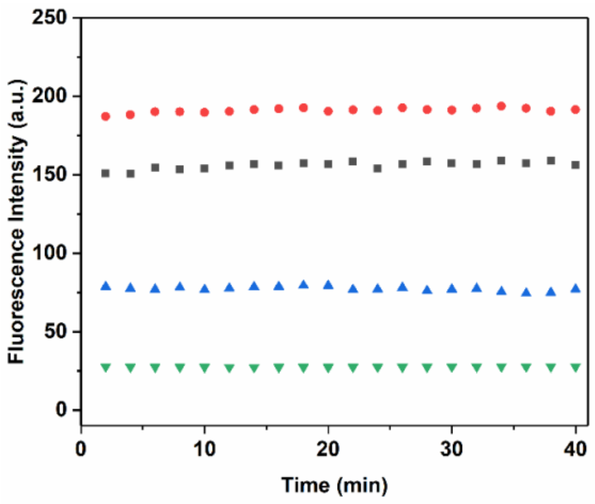

Figure S1. Fluorescence emission of various anomeric duplexes in the presence of ethidium bromide plotted against irradiation time. The EB concentration was 8.5 µM and the duplex concentration was 5 µM. ODN-**5**•ODN-**3** plus EB (black), ODN-**5**•ODN-**6** plus EB (red), ODN-**9**•ODN-**10** plus EB (green), ODN-**5**•ODN-**10** plus EB (blue).

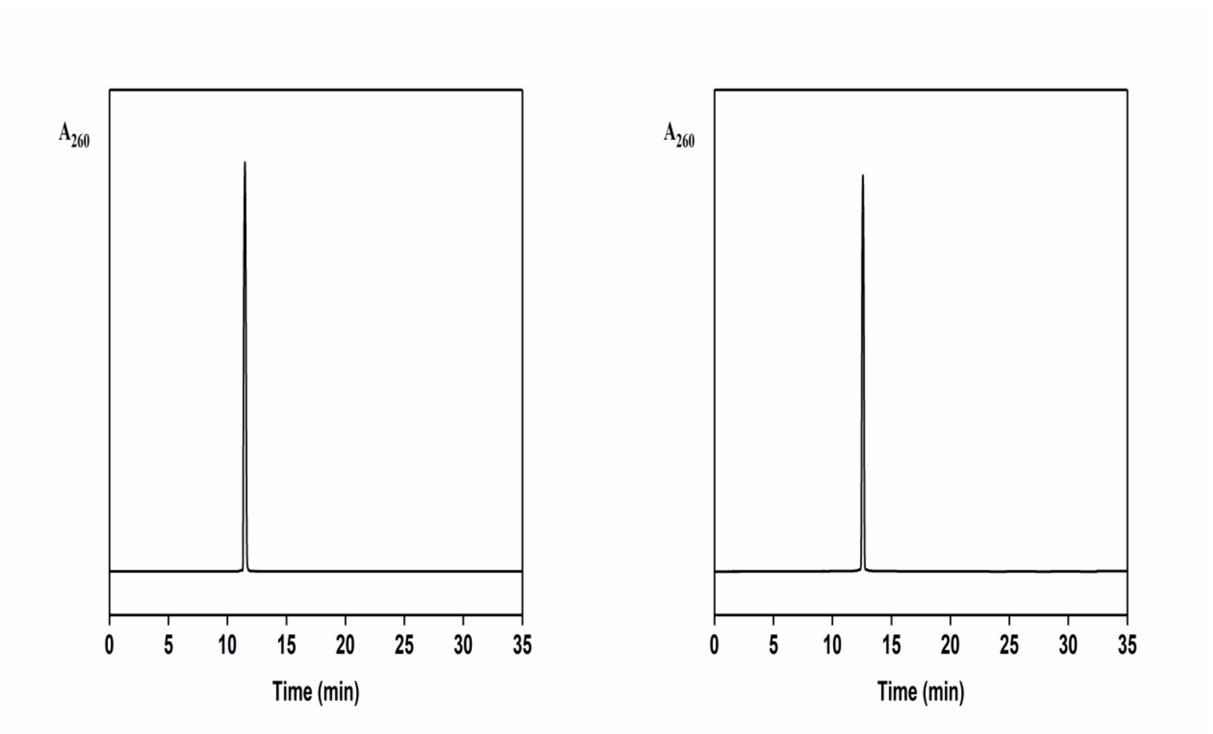

β-5'-d(CAG TTA TGA) (ODN-**3**) β-5'-d(ATC CAG TTA) (ODN-**4**)

Figure S2. Reversed-phase (RP-18) HPLC elution profiles of purified oligonucleotides.

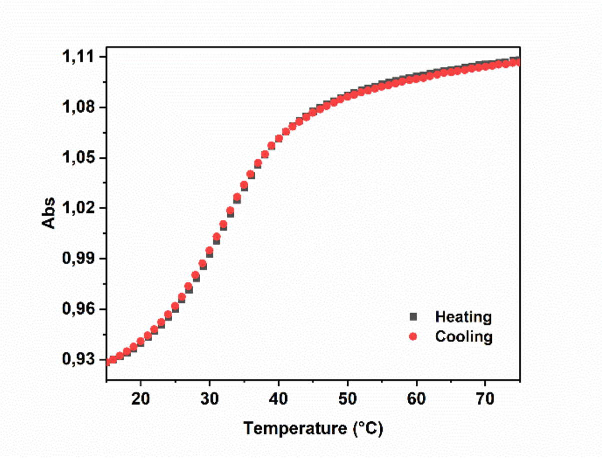

β-5’-d(TCA TAA CTG GAT) (ODN-**5**)
β-3’-d(AGT ATT GAC ……) (ODN-**3**)

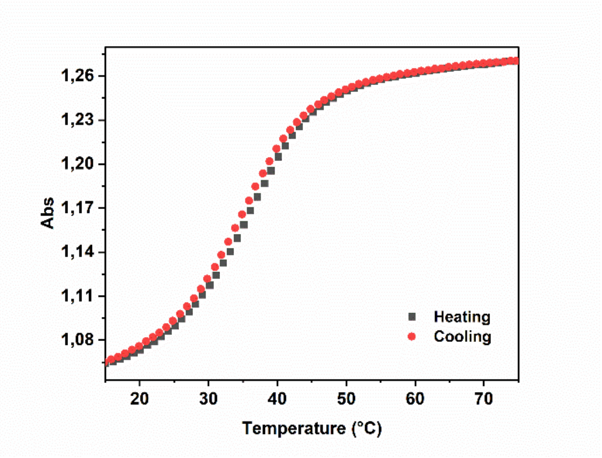

β-5’-d(TCA TAA CTG GAT) (ODN-**5**)*
β-3’-d(AGT ATT GAC ……) (ODN-**3**)

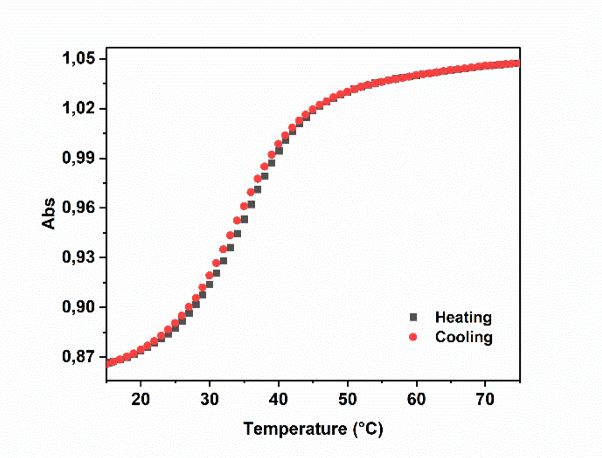

β-5’-d(TCA TAA CTG GAT) (ODN-**5**)
β-3’-d(…….ATT GAC CTA) (ODN-**4**)

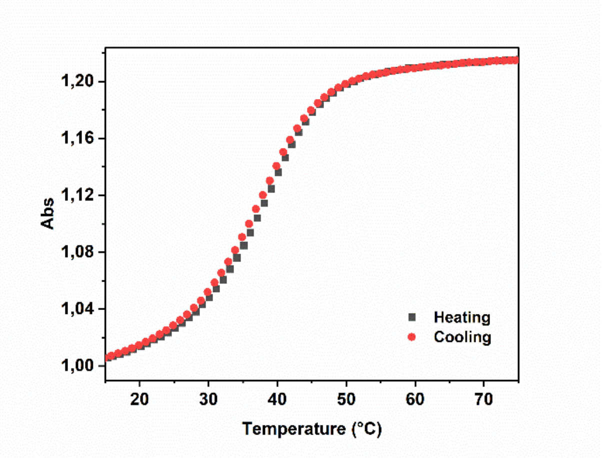

β-5’-d(TCA TAA CTG GAT) (ODN-**5**)*
β-3’-d(…….ATT GAC CTA) (ODN-**4**)

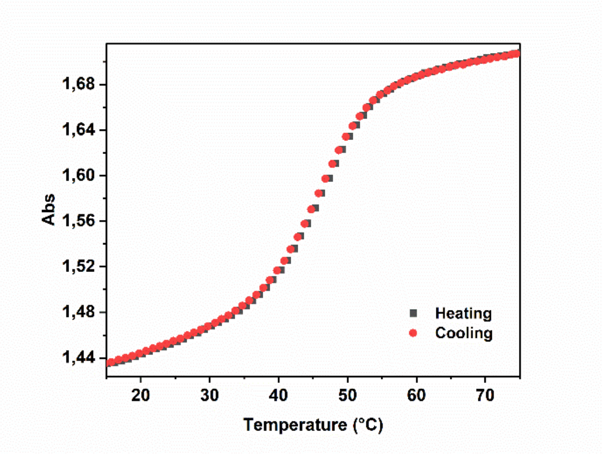

β -5’-d(TCA TAA CTG GAT) (ODN-**5**)
α-5’-d(AGT ATT GAC CTA) (ODN-**10**)

β-3’-d(AGT ATT GAC) (ODN-**3**)

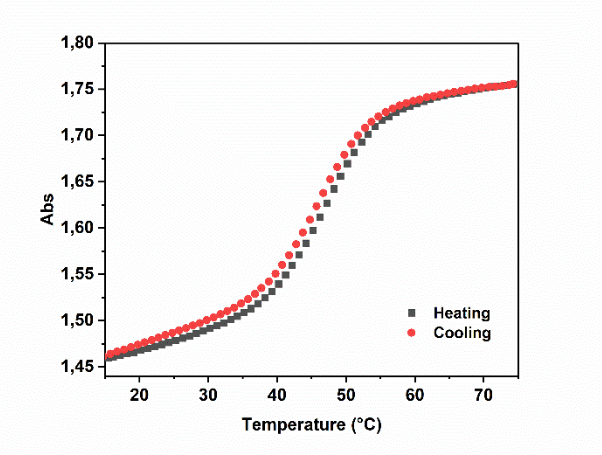

β-5’-d(TCA TAA CTG GAT) (ODN-**5**)
α-5’-d(AGT ATT GAC CTA) (ODN-**10**)

β-3’-d(ATT GAC CTA) (ODN-**4**)

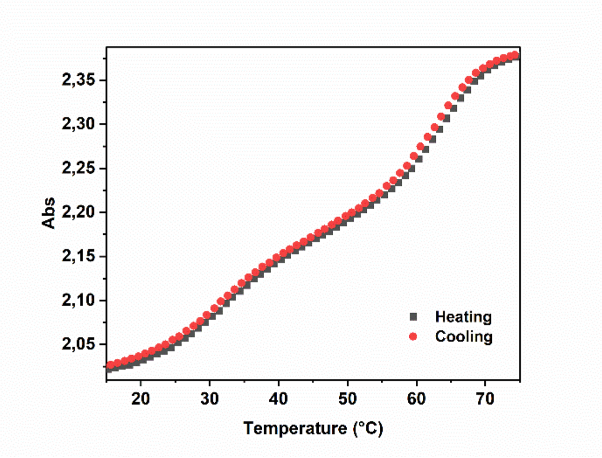

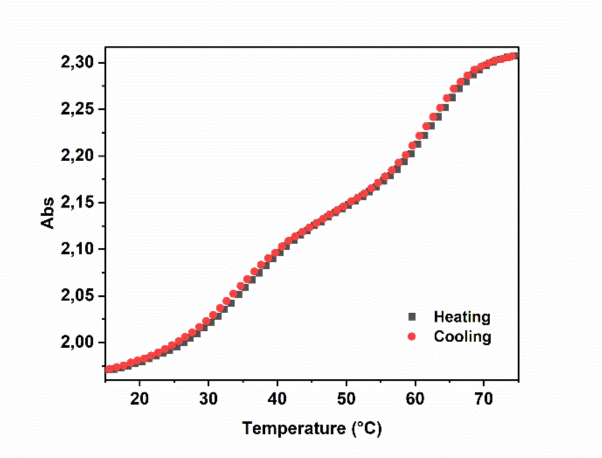

β-5’-d(TCA TAA CTG **GAT**) (ODN-**5**)
β-3’-d(AGT ATT GAC…….) (ODN-**3**)

α-5’-d(TAG GTC AAT ACT) (ODN-**9**)
α-3’-d(ATC CAG TTA TGA) (ODN-**10**)

β-5’-d(**TCA** TAA CTG GAT) (ODN-**5**)
β-3’-d(…… ATT GAC CTA) (ODN-**4**)

α-5’-d(TAG GTC AAT ACT) (ODN-**9**)
α-3’-d(ATC CAG TTA TGA) (ODN-**10**)

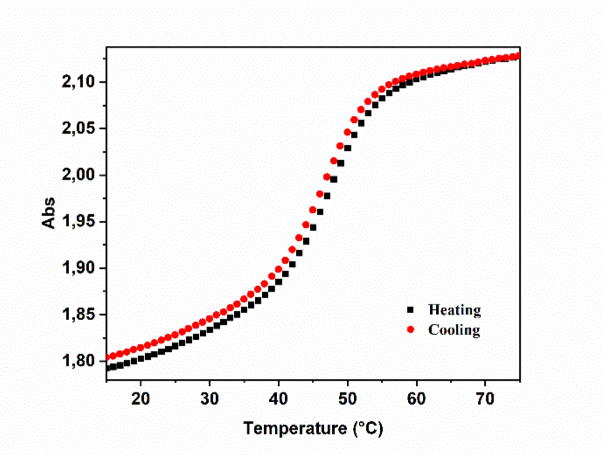

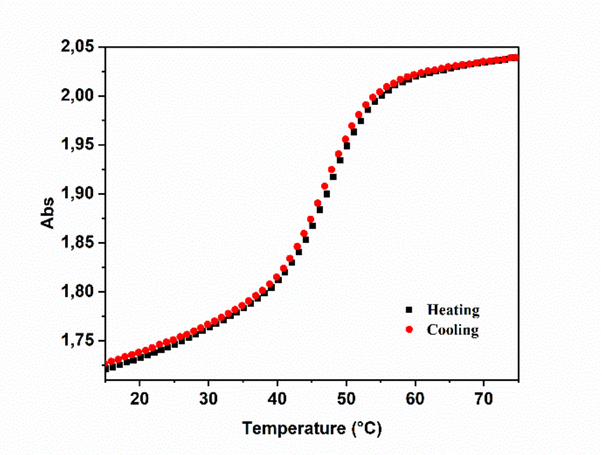

β-5’-d(TCA TAA CTG GAT) (ODN-**5**)
β-3’-d(AGT ATT GAC CTA) (ODN-**6**)

β-3’-d(AGT ATT GAC) (ODN-**3**)

β-5’-d(TCA TAA CTG GAT) (ODN-**5**)
β-3’-d(AGT ATT GAC CTA) (ODN-**6**)

β-3’-d(ATT GAC CTA) (ODN-**4**)

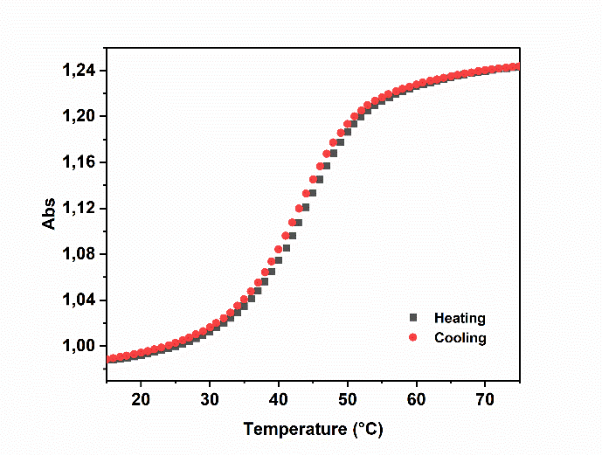

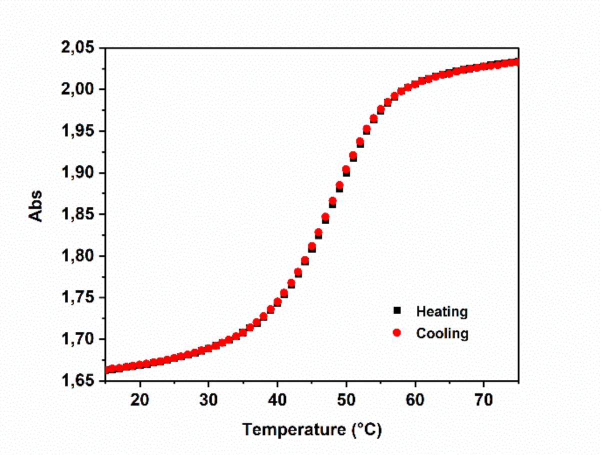

α-5’-d(TCA TAA CTG GAT) (ODN-**7**)
β-5’-d(AGT ATT GAC CTA) (ODN-**2**)

β-5’-d(TAG GTC AAT ACT) (ODN-**1**)
β-3’-d(ATC CAG TTA TGA) (ODN-**2**)

α-5’-d(TCA TAA CTG GAT) (ODN-**7**)

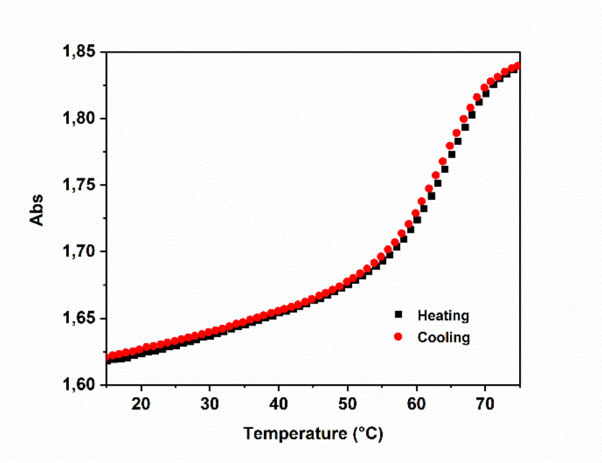

α-5’-d(TCA TAA CTG GAT) (ODN-**7**)
α-3’-d(ATC CAG TTA TGA) (ODN-**8**)

β-5’-d(AGT ATT GAC CTA) (ODN-**2**)

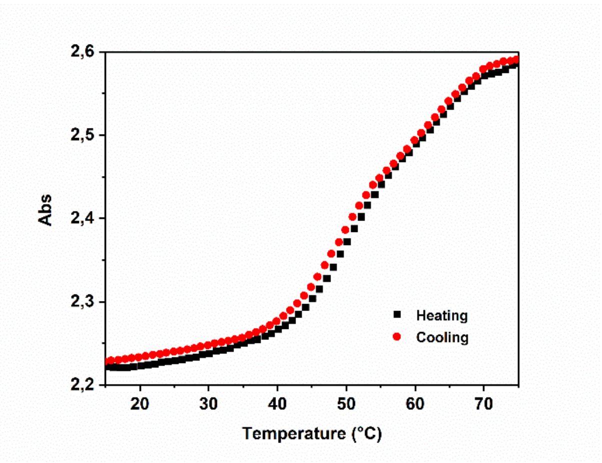

α-5’-d(TCA TAA CTG GAT) (ODN-**7**)
α-3’-d(ATC CAG TTA TGA) (ODN-**8**)

β-5’-d(TAG GTC AAT ACT) (ODN-**1**)
β-3’-d(ATC CAG TTA TGA) (ODN-**2**)

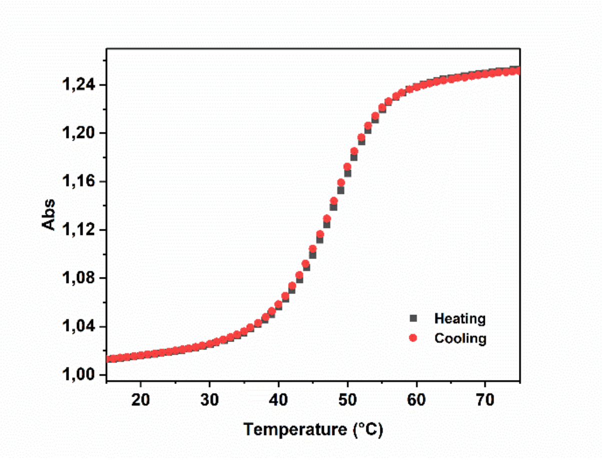

β-5’-d(TAG GTC AAT ACT) (ODN-**1**)
β-3’-d(ATC CAG TTA TGA) (ODN-**2**)

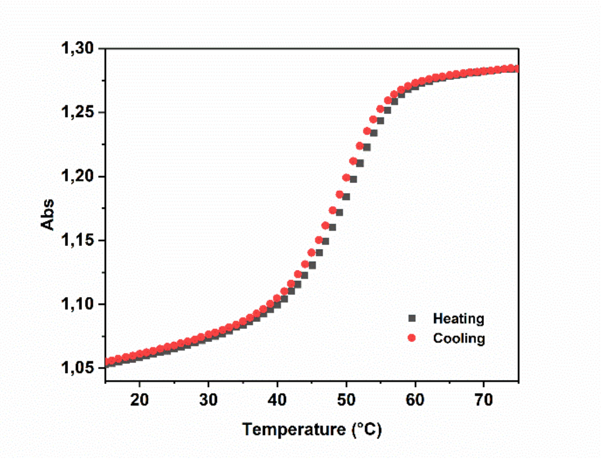

β-5’-d(TAG GTC AAT ACT) (ODN-**1**)*
β-3’-d(ATC CAG TTA TGA) (ODN-**2**)

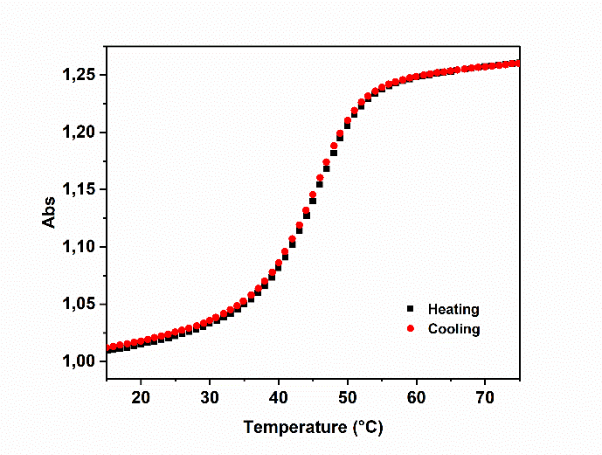

β-5’-d(TCA TAA CTG GAT) (ODN-**5**)
β-3’-d(AGT ATT GAC CTA) (ODN-**6**)

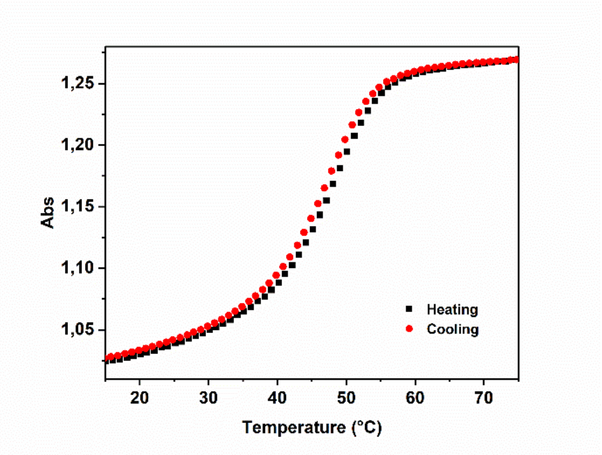

β-5’-d(TCA TAA CTG GAT) (ODN-**5**)*
β-3’-d(AGT ATT GAC CTA) (ODN-**6**)

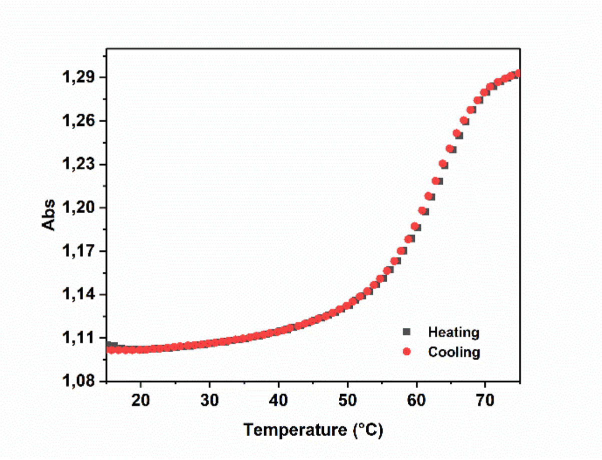

α-5’-d(TCA TAA CTG GAT) (ODN-**7**)
α-3’-d(ATC CAG TTA TGA) (ODN-**8**)

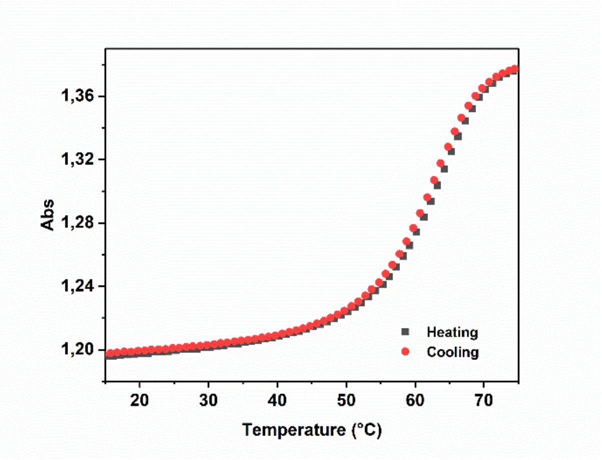

α-5’-d(TCA TAA CTG GAT) (ODN-**7**)*
α-3’-d(ATC CAG TTA TGA) (ODN-**8**)

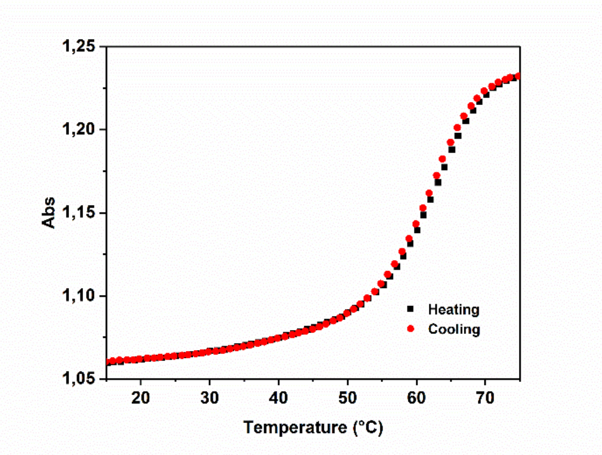

α-5’-d(TAG GTC AAT ACT) (ODN-**9**)
α-3’-d(ATC CAG TTA TGA) (ODN-**10**)

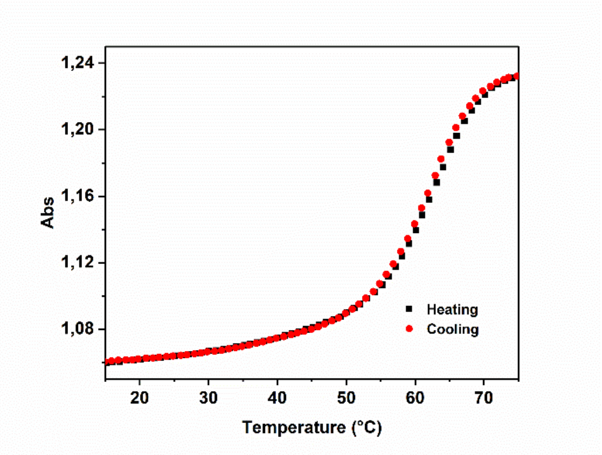

α-5’-d(TAG GTC AAT ACT) (ODN-**9**)*
α-3’-d(ATC CAG TTA TGA) (ODN-**10**)

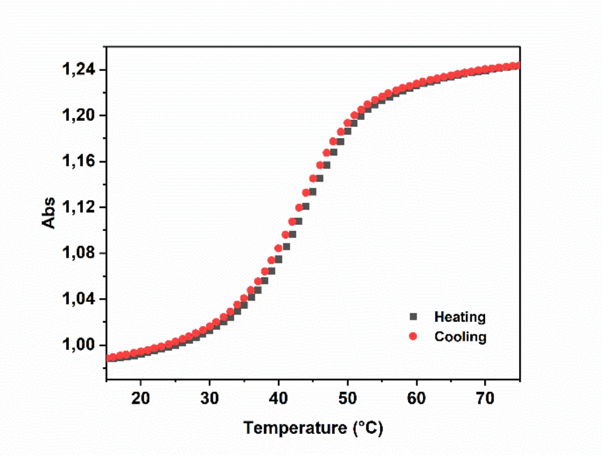

α-5’-d(TCA TAA CTG GAT) (ODN-**7**)
β-5’-d(AGT ATT GAC CTA) (ODN-**2**)

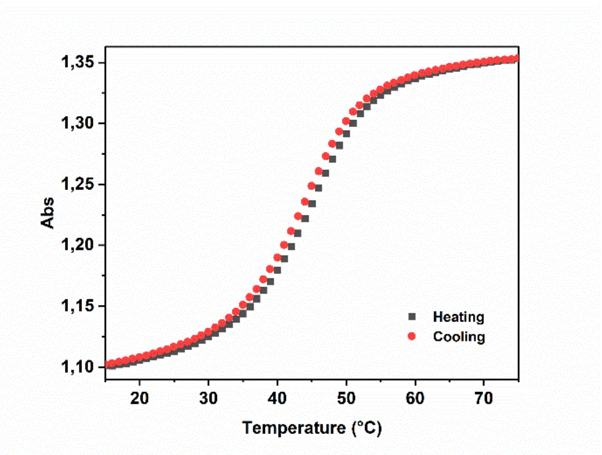

α-5’-d(TCA TAA CTG GAT) (ODN-**7**) *
β-5’-d(AGT ATT GAC CTA) (ODN-**2**)

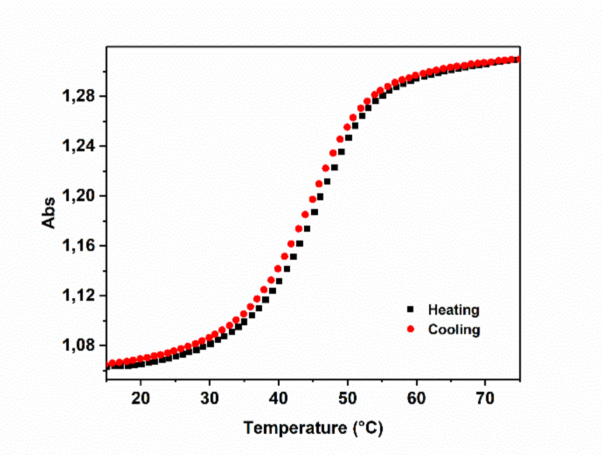

β-5’-d(TAG GTC AAT ACT) (ODN-**1**)
α-5’-d(ATC CAG TTA TGA) (ODN-**8**)

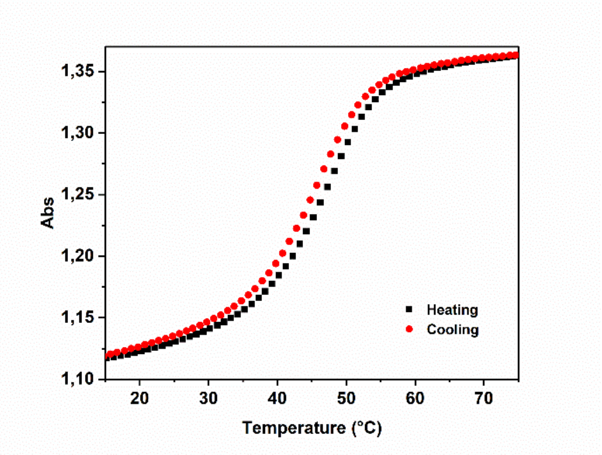

β-5’-d(TAG GTC AAT ACT) (ODN-**1**)*
α-5’-d(ATC CAG TTA TGA) (ODN-**8**)

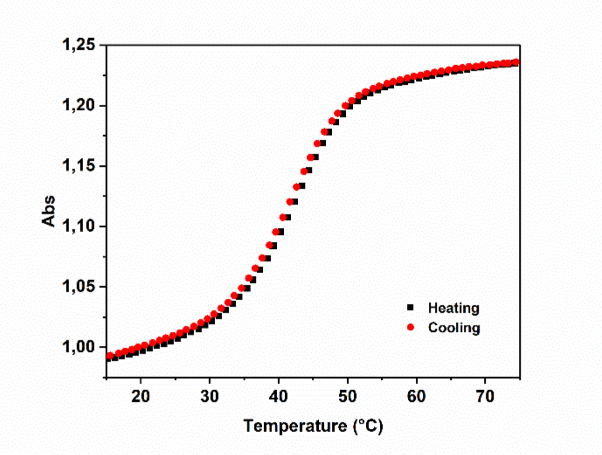

α-5’-d(TAG GTC AAT ACT) (ODN-**9**)
β-5’-d(ATC CAG TTA TGA) (ODN-**6**)

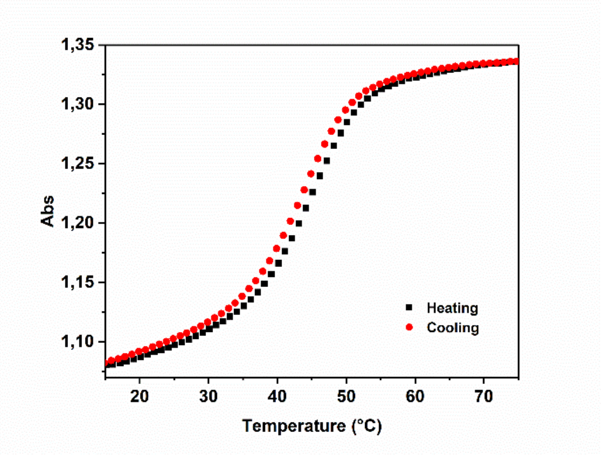

α-5’-d(TAG GTC AAT ACT) (ODN-**9**)*
β-5’-d(ATC CAG TTA TGA) (ODN-**6**)

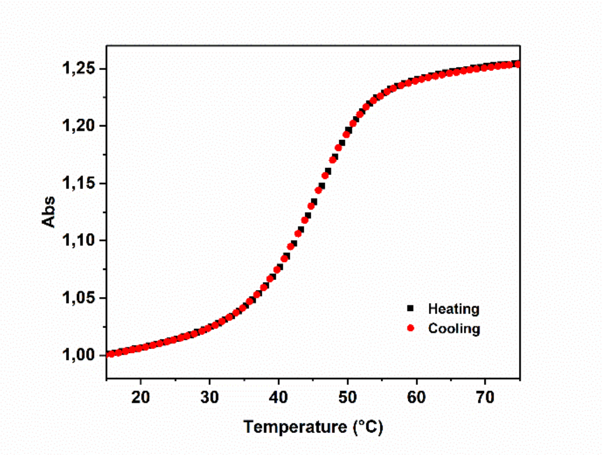

β-5’-d(TCA TAA CTG GAT) (ODN-**5**)
α-5’-d(AGT ATT GAC CTA) (ODN-**10**)

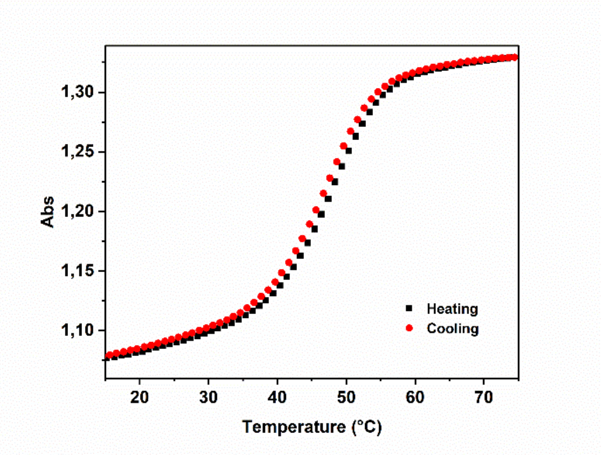

β-5’-d(TCA TAA CTG GAT) (ODN-**5**)*
α-5’-d(AGT ATT GAC CTA) (ODN-**10**)

Figure S3. Thermal denaturation curves obtained from heating (black) and cooling (red) experiments of duplexes monitored at 260 nm. All experiments were performed with 5 µM duplex concentration at a heating rate of 1.0 °C/min in 100 mM NaCl, 10 mM $MgCl_2$, 10 mM Na-cacodylate (pH 7.0) buffer. * In the presence of ethidium bromide.

a)

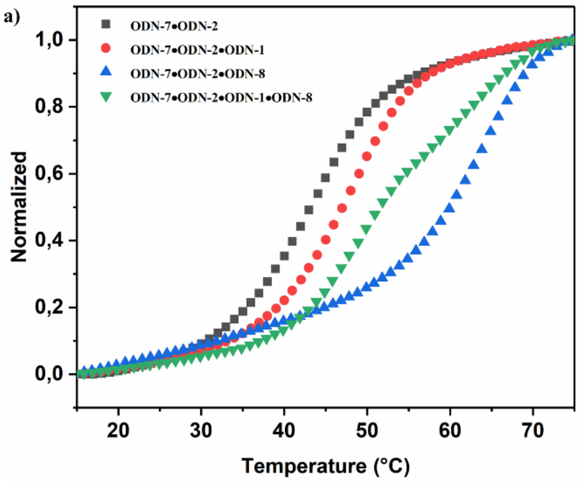

α-5’-d(TCA TAA CTG GAT) (ODN-**7**)
β-5’-d(AGT ATT GAC CTA) (ODN-**2**)

β-5’-d(TAG GTC AAT ACT) (ODN-**1**)
β-3’-d(ATC CAG TTA TGA) (ODN-**2**)
α-5’-d(TCA TAA CTG GAT) (ODN-**7**)

α-5’-d(TCA TAA CTG GAT) (ODN-**7**)
α-3’-d(AGT ATT GAC CTA) (ODN-**8**)
β-5’-d(AGT ATT GAC CTA) (ODN-**2**)

β-5’-d(TAG GTC AAT ACT) (ODN-**1**)
β-3’-d(ATC CAG TTA TGA) (ODN-**2**)
α-5’-d(TCA TAA CTG GAT) (ODN-**7**)
α-3’-d(AGT ATT GAC CTA) (ODN-**8**)

b)

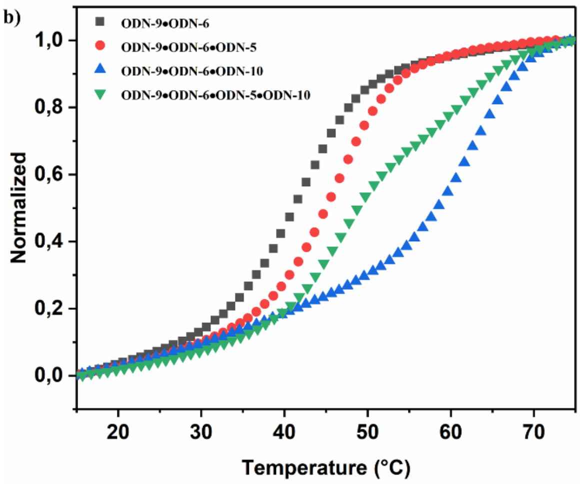

α-5’-d(TAG GTC AAT ACT) (ODN-**9**)
β-5’-d(ACT CAG TTA TGA) (ODN-**6**)

β-5’-d(TCA TAA CTG GAT) (ODN-**5**)
β-3’-d(AGT ATT GAC TCA) (ODN-**6**)
α-5’-d(TAG GTC AAT ACT) (ODN-**9**)

α-5’-d(TAG GTC AAT ACT) (ODN-**9**)
α-3’-d(ATC CAG TTA TGA) (ODN-**10**)
β-5’-d(ACT CAG TTA TGA) (ODN-**6**)

β-5’-d(TCA TAA CTG GAT) (ODN-**5**)
β-3’-d(AGT ATT GAC TCA) (ODN-**6**)
α-5’-d(TAG GTC AAT ACT) (ODN-**9**)
α-3’-d(ATC CAG TTA TGA) (ODN-**10**)

c)

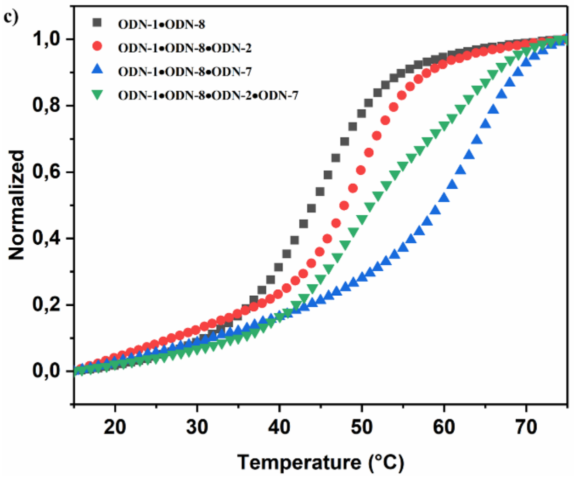

β-5’-d(TAG GTC AAT ACT) (ODN-**1**)
α-5’-d(ATC CAG TTA TGA) (ODN-**8**)

β-5’-d(TAG GTC AAT ACT) (ODN-**1**)
β-3’-d(ATC CAG TTA TGA) (ODN-**2**)
α-5’-d(ATC CAG TTA TGA) (ODN-**8**)

α-5’-d(TCA TAA CTG GAT) (ODN-**7**)

d)

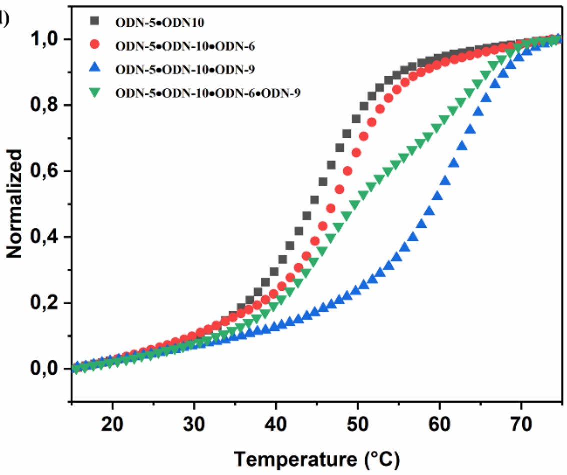

β-5’-d(TCA TAA CTG GAT) (ODN-**5**)
α-5’-d(AGT ATT GAC CTA) (ODN-**10**)

β-5’-d(TCA TAA CTG GAT) (ODN-**5**)
β-3’-d(AGT ATT GAC TCA) (ODN-**6**)
α-5’-d(AGT ATT GAC CTA) (ODN-**10**)

α-5’-d(TAG GTC AAT ACT) (ODN-**9**)

α-3’-d(AGT ATT GAC CTA) (ODN-**8**)
β-5’-d(TAG GTC AAT ACT) (ODN-**1**)

α-3’-d(ATC CAG TTA TGA) (ODN-**10**)
β-5’-d(TCA TAA CTG GAT) (ODN-**5**)

β-5’-d(TAG GTC AAT ACT) (ODN-**1**)
β-3’-d(ATC CAG TTA TGA) (ODN-**2**)
α-5’-d(TCA TAA CTG GAT) (ODN-**7**)
α-3’-d(AGT ATT GAC CTA) (ODN-**8**)

β-5’-d(TCA TAA CTG GAT) (ODN-**5**)
β-3’-d(AGT ATT GAC TCA) (ODN-**6**)
α-5’-d(TAG GTC AAT ACT) (ODN-**9**)
α-3’-d(ATC CAG TTA TGA) (ODN-**10**)

Figure S4. Thermal denaturation experiments of α/β parallel stranded duplexes plus β-D or α-D invader strands. a) ODN-**7**·ODN-**2** (black), ODN-**7**·ODN-**2**·ODN-**1** (red), ODN-**7**·ODN-**2**·ODN-**8** (blue), and ODN-**7**·ODN-**2**·ODN-**1**·ODN-**8** (green); b) ODN-**9**·ODN-**6** (black), ODN-**9**·ODN-**6**·ODN-**5** (red), ODN-**9**·ODN-**6**·ODN-**10** (blue), and ODN-**9**·ODN-**6**·ODN-**5**·ODN-**10** (green); c) ODN-**1**·ODN-**8** (black), ODN-**1**·ODN-**8**·ODN-**2** (red), ODN-**1**·ODN-**8**·ODN-**7** (blue), and ODN-**1**·ODN-**8**·ODN-**2**·ODN-**7** (green); d) ODN-**5**·ODN-**10** (black), ODN-**5**·ODN-**10**·ODN-**6** (red), ODN-**5**·ODN-**10**·ODN-**9** (blue), and ODN-**5**·ODN-**10**·ODN-**6**·ODN-**9** (green). All measurements were performed at 260 nm with 5 µM+5 µM single-strand concentration in 100 mM NaCl, 10 mM $MgCl_2$, and 10 mM Na-cacodylate (pH 7.0).

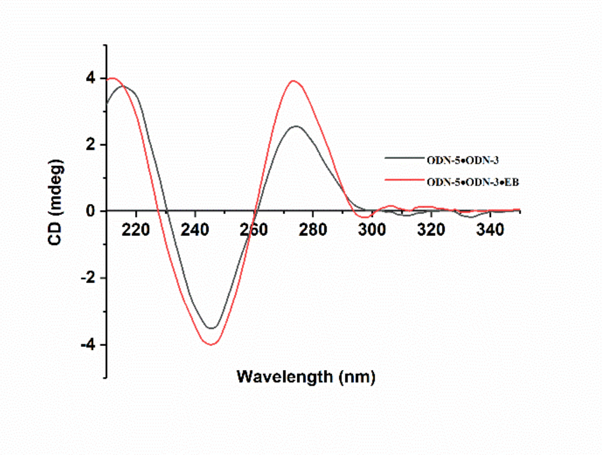

β-5’-d(TCA TAA CTG GAT) (ODN-**5**)
β-3’-d(AGT ATT GAC…….) (ODN-**3**)

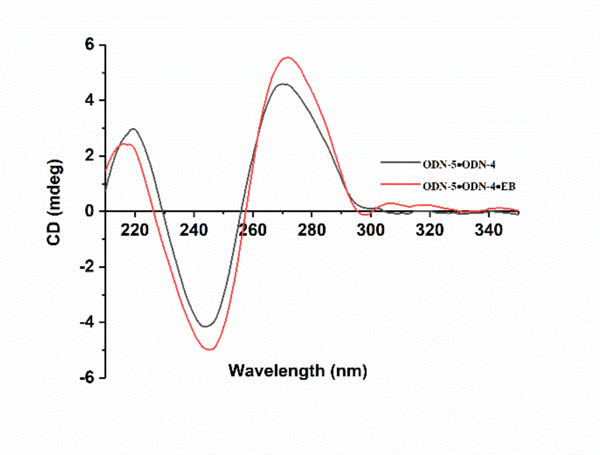

β-5’-d(TCA TAA CTG GAT) (ODN-**5**)
β-3’-d(…….ATT GAC CTA) (ODN-**4**)

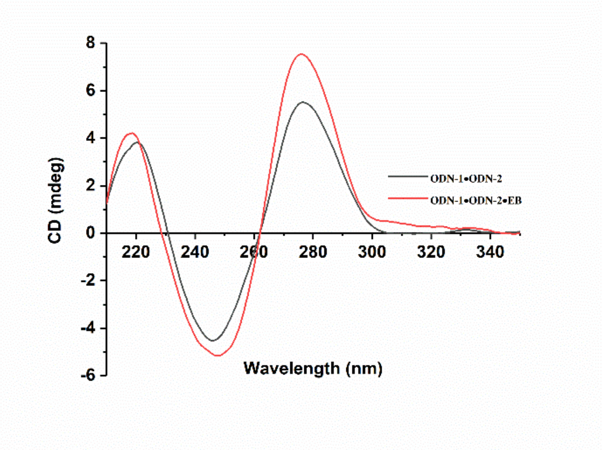

β-5’-d(TAG GTC AAT ACT) (ODN-**1**)
β-3’-d(ATC CAG TTA TGA) (ODN-**2**)

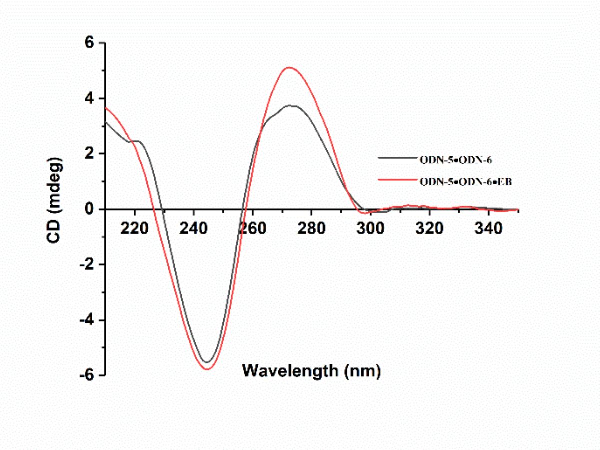

β-5’-d(TCA TAA CTG GAT) (ODN-**5**)
β-3’-d(AGT ATT GAC CTA) (ODN-**6**)

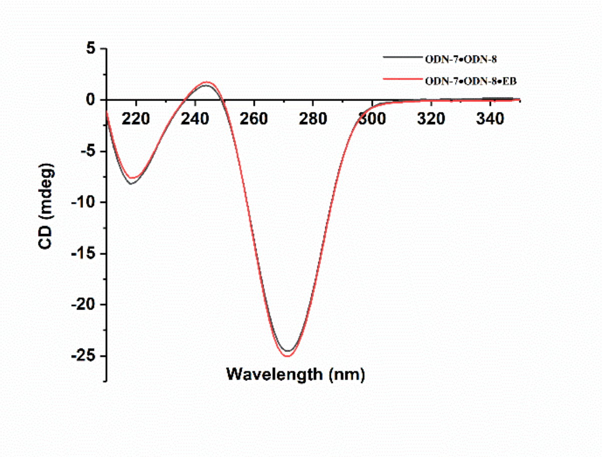

α-5’-d(TCA TAA CTG GAT) (ODN-**7**)
α-3’-d(ATC CAG TTA TGA) (ODN-**8**)

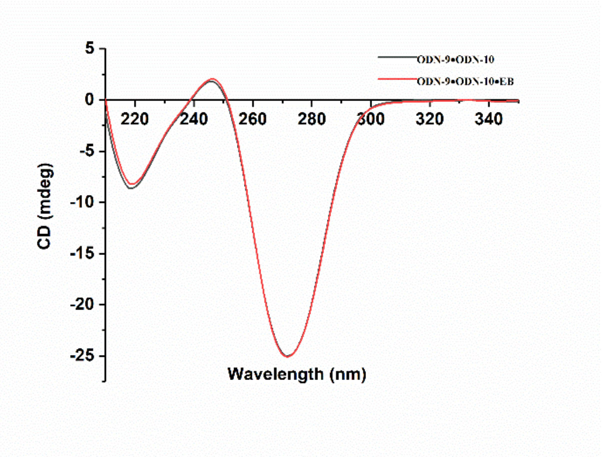

α-5’-d(TAG GTC AAT ACT) (ODN-**9**)
α-3’-d(ATC CAG TTA TGA) (ODN-**10**)

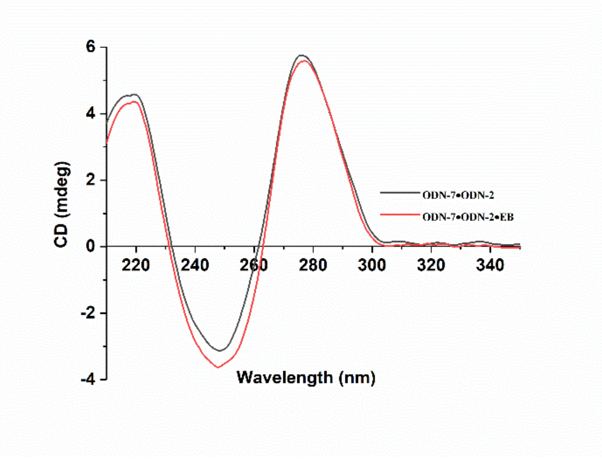

α-5’-d(TCA TAA CTG GAT) (ODN-**7**)
β-5’-d(AGT ATT GAC CTA) (ODN-**2**)

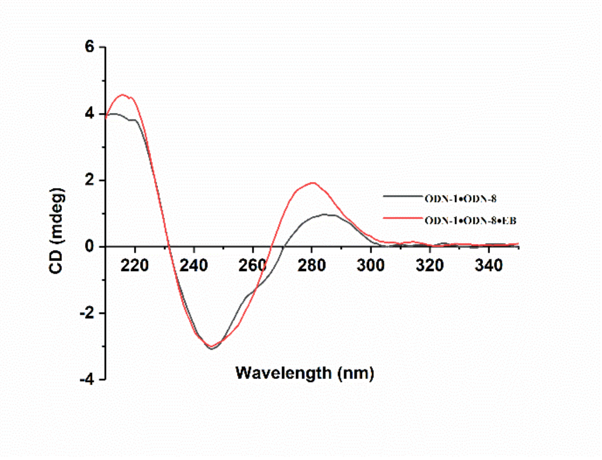

β-5’-d(TAG GTC AAT ACT) (ODN-**1**)
α-5’-d(ATC CAG TTA TGA) (ODN-**8**)

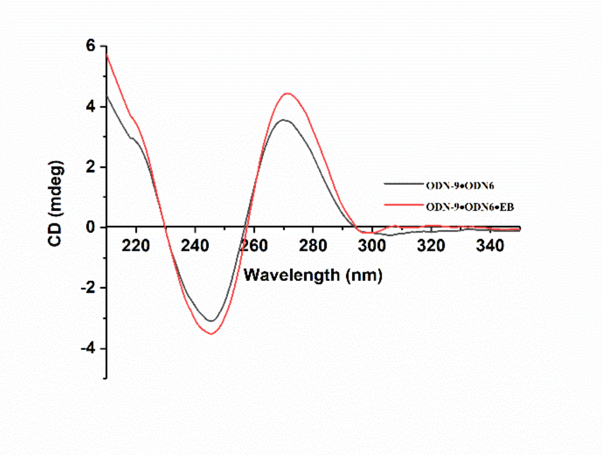

α-5’-d(TAG GTC AAT ACT) (ODN-**9**)
β-5’-d(ATC CAG TTA TGA) (ODN-**6**)

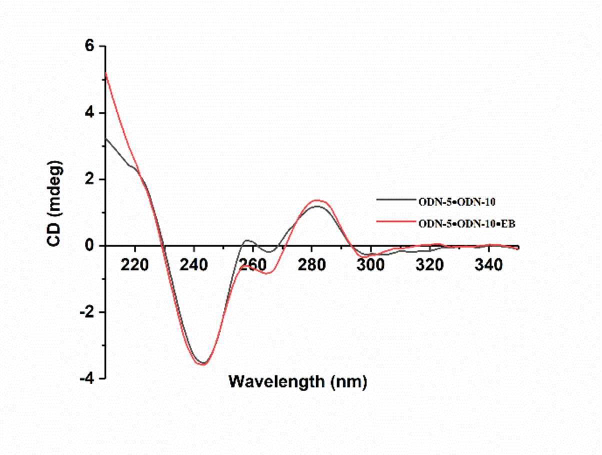

β-5’-d(TCA TAA CTG GAT) (ODN-**5**)
α-5’-d(AGT ATT GAC CTA) (ODN-**10**)

Figure S5. CD-spectra of oligonucleotide duplexes in the absence and presence of ethidium bromide. All measurements were performed in 100 mM NaCl, 10 mM $MgCl_2$, 10 mM Na-cacodylate, pH 7.0 with 5 µM duplex and 8.5 µM EB. The cell path length of the cuvette for the CD-spectra was 5 mm.

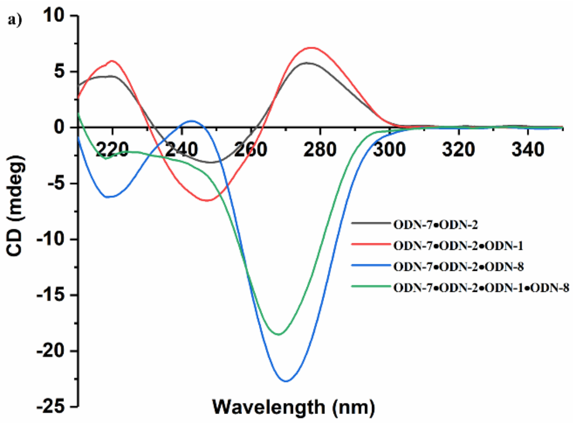

α-5’-d(TCA TAA CTG GAT) (ODN-**7**)
β-5’-d(AGT ATT GAC CTA) (ODN-**2**)

β-5’-d(TAG GTC AAT ACT) (ODN-**1**)
β-3’-d(ATC CAG TTA TGA) (ODN-**2**)
α-5’-d(TCA TAA CTG GAT) (ODN-**7**)

α-5’-d(TCA TAA CTG GAT) (ODN-**7**)
α-3’-d(AGT ATT GAC CTA) (ODN-**8**)
β-5’-d(AGT ATT GAC CTA) (ODN-**2**)

β-5’-d(TAG GTC AAT ACT) (ODN-**1**)
β-3’-d(ATC CAG TTA TGA) (ODN-**2**)
α-5’-d(TCA TAA CTG GAT) (ODN-**7**)
α-3’-d(AGT ATT GAC CTA) (ODN-**8**)

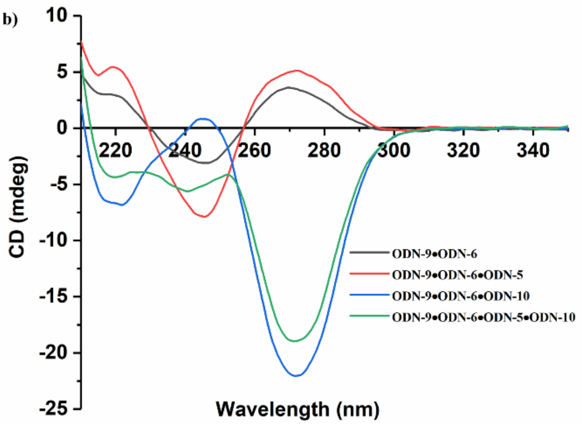

α-5’-d(TAG GTC AAT ACT) (ODN-**9**)
β-5’-d(ACT CAG TTA TGA) (ODN-**6**)

β-5’-d(TCA TAA CTG GAT) (ODN-**5**)
β-3’-d(AGT ATT GAC TCA) (ODN-**6**)
α-5’-d(TAG GTC AAT ACT) (ODN-**9**)

α-5’-d(TAG GTC AAT ACT) (ODN-**9**)
α-3’-d(ATC CAG TTA TGA) (ODN-**10**)
β-5’-d(ACT CAG TTA TGA) (ODN-**6**)

β-5’-d(TCA TAA CTG GAT) (ODN-**5**)
β-3’-d(AGT ATT GAC TCA) (ODN-**6**)
α-5’-d(TAG GTC AAT ACT) (ODN-**9**)
α-3’-d(ATC CAG TTA TGA) (ODN-**10**)

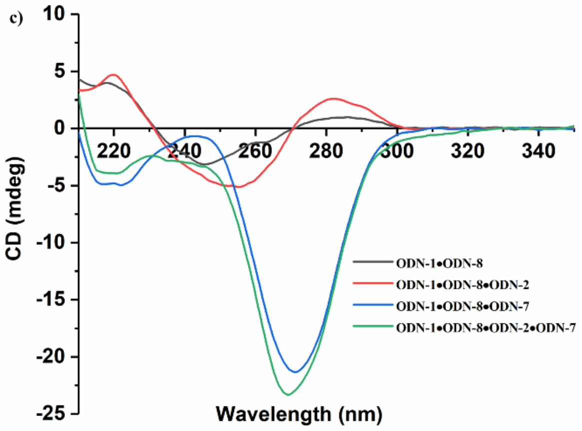

β-5’-d(TAG GTC AAT ACT) (ODN-**1**)
α-5’-d(ATC CAG TTA TGA) (ODN-**8**)

β-5’-d(TAG GTC AAT ACT) (ODN-**1**)
β-3’-d(ATC CAG TTA TGA) (ODN-**2**)
α-5’-d(ATC CAG TTA TGA) (ODN-**8**)

α-5’-d(TCA TAA CTG GAT) (ODN-**7**)
α-3’-d(AGT ATT GAC CTA) (ODN-**8**)
β-5’-d(TAG GTC AAT ACT) (ODN-**1**)

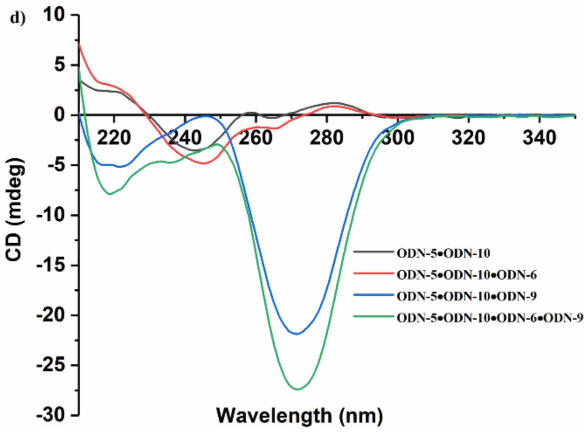

β-5’-d(TCA TAA CTG GAT) (ODN-**5**)
α-5’-d(AGT ATT GAC CTA) (ODN-**10**)

β-5’-d(TCA TAA CTG GAT) (ODN-**5**)
β-3’-d(AGT ATT GAC TCA) (ODN-**6**)
α-5’-d(AGT ATT GAC CTA) (ODN-**10**)

α-5’-d(TAG GTC AAT ACT) (ODN-**9**)
α-3’-d(ATC CAG TTA TGA) (ODN-**10**)
β-5’-d(TCA TAA CTG GAT) (ODN-**5**)

β-5’-d(TAG GTC AAT ACT) (ODN-**1**)
β-3’-d(ATC CAG TTA TGA) (ODN-**2**)
α-5’-d(TCA TAA CTG GAT) (ODN-**7**)
α-3’-d(AGT ATT GAC CTA) (ODN-**8**)

β-5’-d(TCA TAA CTG GAT) (ODN-**5**)
β-3’-d(AGT ATT GAC TCA) (ODN-**6**)
α-5’-d(TAG GTC AAT ACT) (ODN-**9**)
α-3’-d(ATC CAG TTA TGA) (ODN-**10**)

Figure S6. CD-spectra of α/β parallel stranded duplexes plus β-D or α-D invader strand. a) ODN-**7**·ODN-**2** (black), ODN-**7**·ODN-**2**·ODN-**1** (red), ODN-**7**·ODN-**2**·ODN-**8** (blue), and ODN-**7**·ODN-**2**·ODN-**1**·ODN-**8** (green); b) ODN-**9**·ODN-**6** (black), ODN-**9**·ODN-**6**·ODN-**5** (red), ODN-**9**·ODN-**6**·ODN-**10** (blue), and ODN-**9**·ODN-**6**·ODN-**5**·ODN-**10** (green); c) ODN-**1**·ODN-**8** (black), ODN-**1**·ODN-**8**·ODN-**2** (red), ODN-**1**·ODN-**8**·ODN-**7** (blue), and ODN-**1**·ODN-**8**·ODN-**2**·ODN-**7** (green); d) ODN-**5**·ODN-**10** (black), ODN-**5**·ODN-**10**·ODN-**6** (red), ODN-**5**·ODN-**10**·ODN-**9** (blue), and ODN-**5**·ODN-**10**·ODN-**6**·ODN-**9** (green). All measurements were performed with 5 µM+5 µM single strand concentration in 100 mM NaCl, 10 mM $MgCl_2$, and 10 mM Na-cacodylate (pH 7.0). The cell path length of the cuvette used to record the CD-spectra was 5 mm.

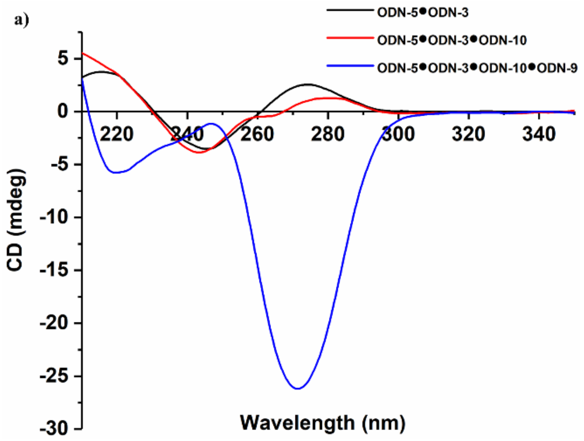

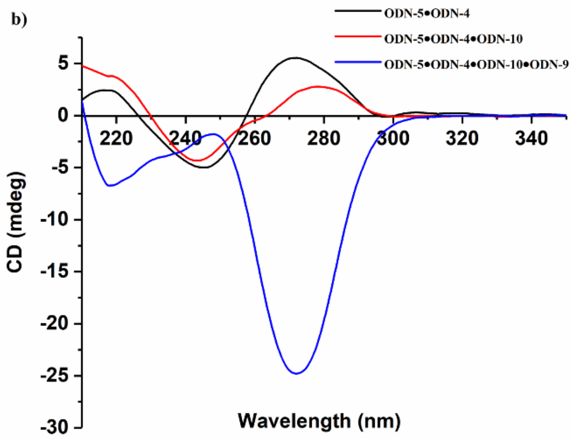

β-5’-d(TCA TAA CTG GAT) (ODN-**5**)
β-3’-d(AGT ATT GAC ……) (ODN-**3**)

β-5’-d(TCA TAA CTG GAT) (ODN-**5**)
α-5’-d(AGT ATT GAC CTA) (ODN-**10)**
β-3’-d(AGT ATT GAC ……) (ODN-**3**)

β-5’-d(TCA TAA CTG GAT) (ODN-**5**)
β-3’-d(AGT ATT GAC ……) (ODN-**3**)
α-5’-d(TAG GTC AAT ACT) (ODN-**9**)
α-3’-d(ATC CAG TTA TGA) (ODN-**10**)

β-5’-d(TCA TAA CTG GAT) (ODN-**5**)
β-3’-d(…….ATT GAC CTA) (ODN-**4**)

β-5’-d(TCA TAA CTG GAT) (ODN-**5**)
α-5’-d(AGT ATT GAC CTA) (ODN-**10)**
β-3’-d(…….ATT GAC CTA) (ODN-**4**)

β-5’-d(TCA TAA CTG GAT) (ODN-**5**)
β-3’-d(…….ATT GAC CTA) (ODN-**4**)
α-5’-d(TAG GTC AAT ACT) (ODN-**9**)
α-3’-d(ATC CAG TTA TGA) (ODN-**10**)

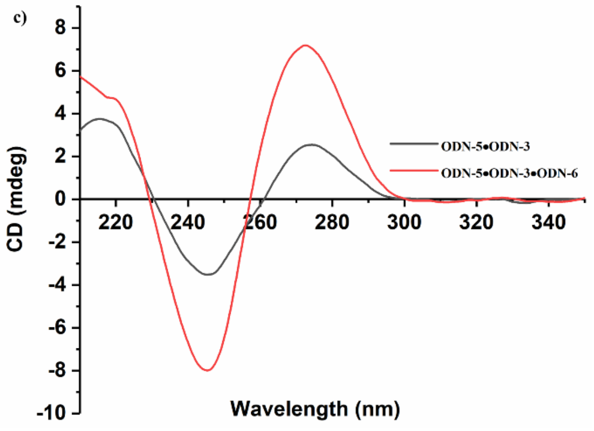

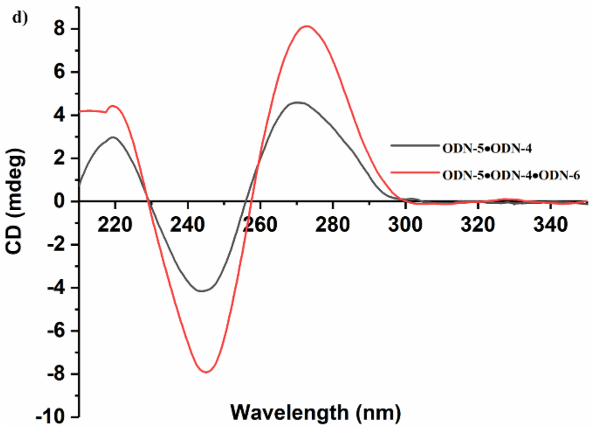

β-5’-d(TCA TAA CTG GAT) (ODN-**5**)
β-3’-d(AGT ATT GAC ……) (ODN-**3**)

β-5’-d(TCA TAA CTG GAT) (ODN-**5**)
β-3’-d(AGT ATT GAC CTA) (ODN-**6)**
β-3’-d(AGT ATT GAC ……) (ODN-**3**)

β-5’-d(TCA TAA CTG GAT) (ODN-**5**)
β-3’-d(……. ATT GAC CTA) (ODN-**4**)

β-5’-d(TCA TAA CTG GAT) (ODN-**5**)
β-3’-d(AGT ATT GAC CTA) (ODN-**6)**
β-3’-d(……. ATT GAC CTA) (ODN-**4**)

Figure S7. CD-spectra of β/β duplexes with toehold plus corresponding α-D or β-D invader strand. a) ODN-**5**·ODN-**3** (black), ODN-**5**·ODN-**3**·ODN-**10** (red), ODN-**5**·ODN-**3**·ODN-**10**·ODN-**9** (blue); b) ODN-**5**·ODN-**4** (black), ODN-**5**·ODN-**4**·ODN-**10** (red), ODN-**5**·ODN-**4**·ODN-**10**·ODN-**9** (blue); c) ODN-**5**·ODN-**3** (black), ODN-**5**·ODN-**3**·ODN-**6** (red); d) ODN-**5**·ODN-**4** (black), ODN-**5**·ODN-**4**·ODN-**6** (red). All measurements were performed with 5 µM + 5 µM single strand concentration in 100 mM NaCl, 10 mM $MgCl_2$, and 10 mM Na-cacodylate (pH 7.0). The cell path length of the cuvette used to record the CD-spectra was 5 mm.

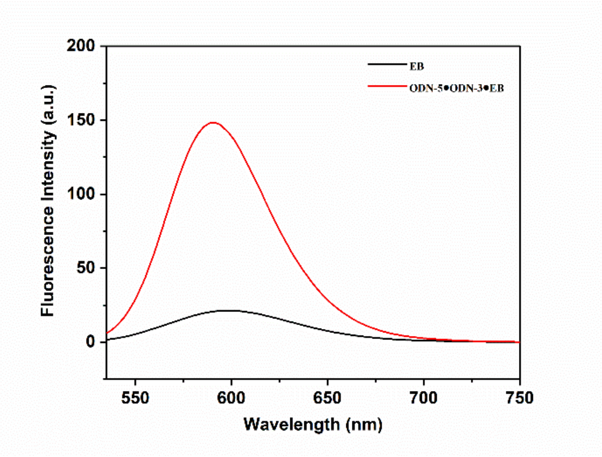

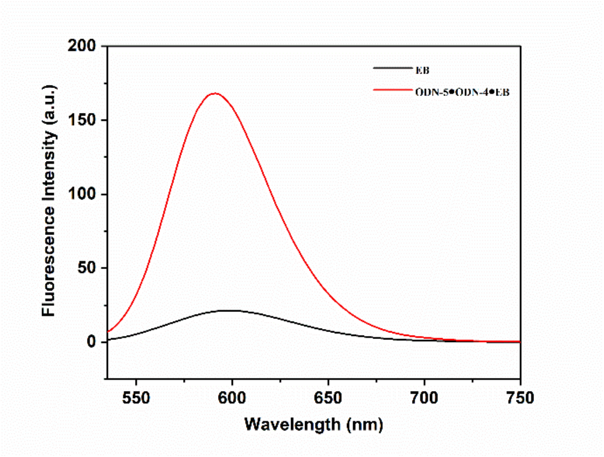

β-5’-d(TCA TAA CTG GAT) (ODN-**5**)
β-3’-d(AGT ATT GAC.……) (ODN-**3**)

β-5’-d(TCA TAA CTG GAT) (ODN-**5**)
β-3’-d(…….ATT GAC CTA) (ODN-**4**)

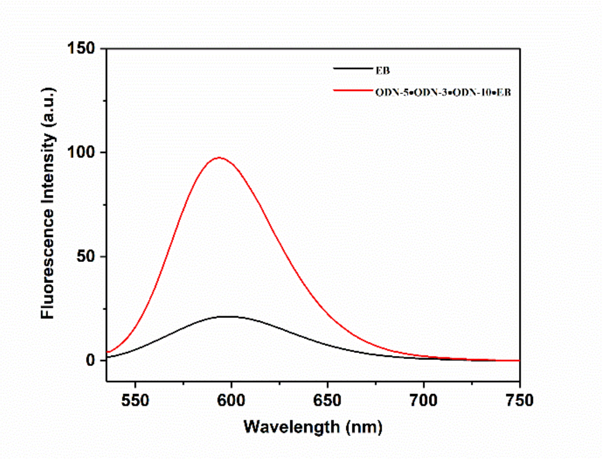

β -5’-d(TCA TAA CTG GAT) (ODN-**5**)
α-5’-d(AGT ATT GAC CTA) (ODN-**10**)

β-3’-d(AGT ATT GAC…….) (ODN-**3**)

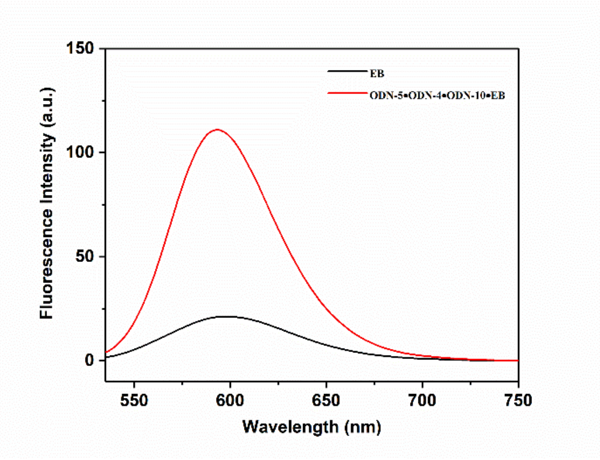

β-5’-d(TCA TAA CTG GAT) (ODN-**5**)
α-5’-d(AGT ATT GAC CTA) (ODN-**10**)

β-3’-d(…….ATT GAC CTA) (ODN-**4**)

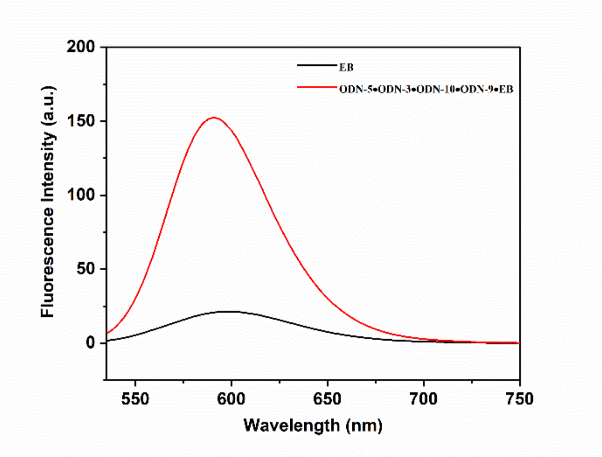

β-5’-d(TCA TAA CTG GAT) (ODN-**5**)
β-3’-d(AGT ATT GAC…….) (ODN-**3**)
α-5’-d(TAG GTC AAT ACT) (ODN-**9**)
α-3’-d(ATC CAG TTA TGA) (ODN-**10**)

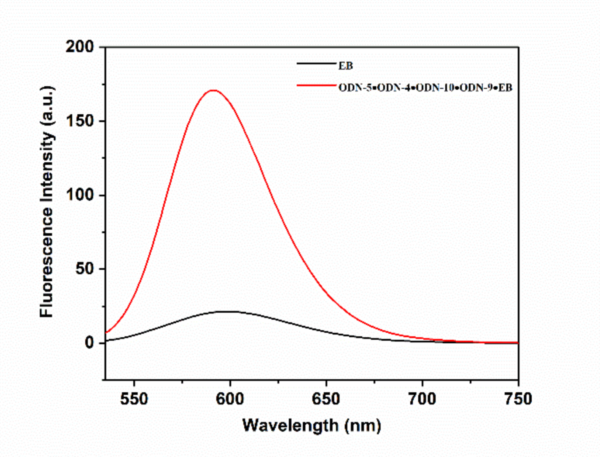

β-5’-d(TCA TAA CTG GAT) (ODN-**5**)
β-3’-d(…… ATT GAC CTA) (ODN-**4**)
α-5’-d(TAG GTC AAT ACT) (ODN-**9**)
α-3’-d(ATC CAG TTA TGA) (ODN-**10**)

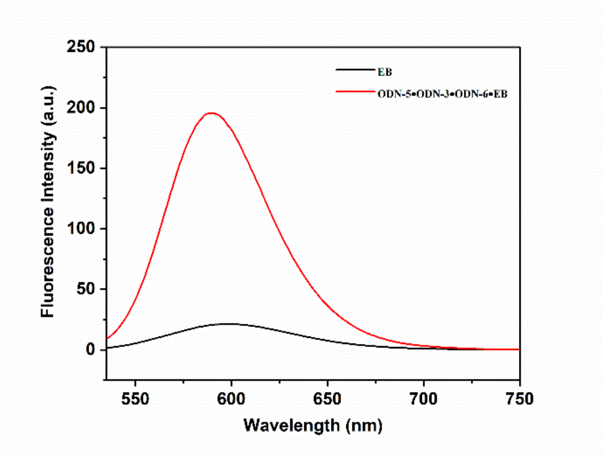

β-5’-d(TCA TAA CTG GAT) (ODN-**5**)
β-3’-d(AGT ATT GAC CTA) (ODN-**6**)

β-3’-d(AGT ATT GAC…….) (ODN-**3**)

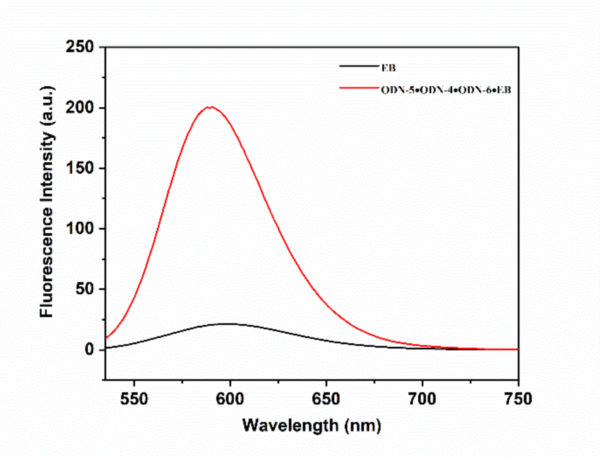

β-5’-d(TCA TAA CTG GAT) (ODN-**5**)
β-3’-d(AGT ATT GAC CTA) (ODN-**6**)

β-3’-d(…….ATT GAC CTA) (ODN-**4**)

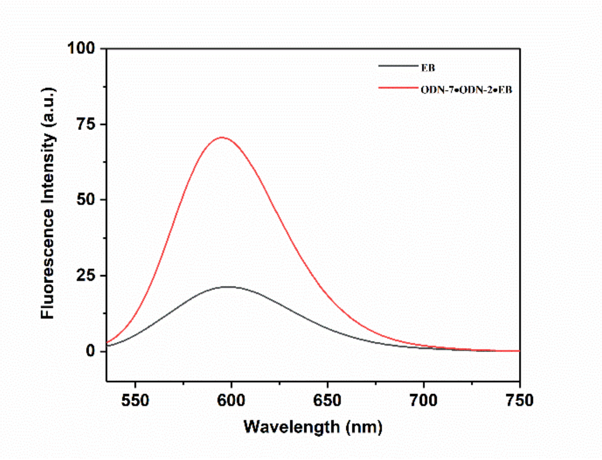

α-5’-d(TCA TAA CTG GAT) (ODN-**7**)
β-5’-d(AGT ATT CAC CTA) (ODN-**2**)

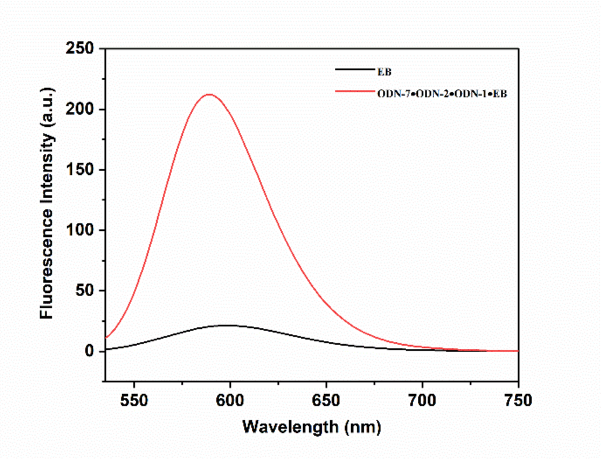

β-5’-d(TAG GTC AAT ACT) (ODN-**1**)
β-3’-d(ATC CAG TTA TGA) (ODN-**2**)

α-5’-d(TCA TAA CTG GAT) (ODN-**7**)

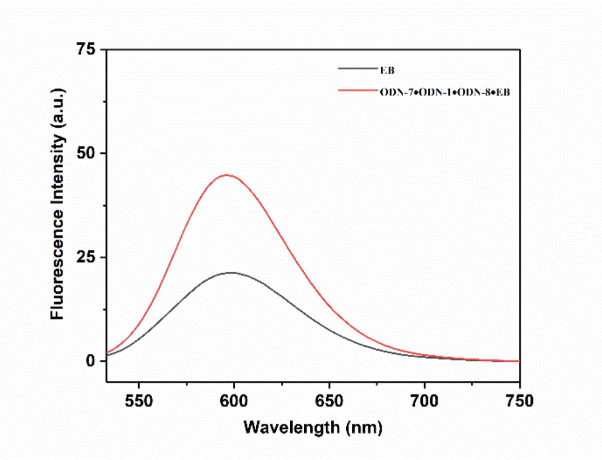

α-5’-d(TCA TAA CTG GAT) (ODN-**7**)
α-3’-d(ATC CAG TTA TGA) (ODN-**8**)

β-5’-d(AGT ATT GAC CTA) (ODN-**2**)

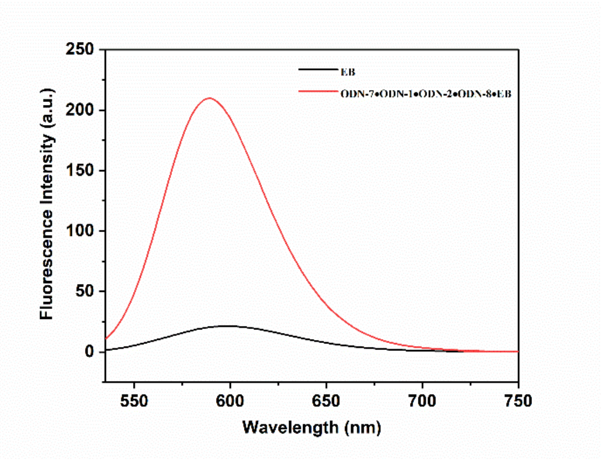

α-5’-d(TCA TAA CTG GAT) (ODN-**7**)
α-3’-d(ATC CAG TTA TGA) (ODN-**8**)

β-5’-d(TAG GTC AAT ACT) (ODN-**1**)
β-3’-d(ATC CAG TTA TGA) (ODN-**2**)

Figure S8. Fluorescence emission spectra of duplexes plus released strand in the presence of ethidium bromide (8.5 µM). All spectra were measured in 100 mM NaCl, 10 mM $MgCl_2$, and 10 mM Na-cacodylate (pH 7.0).

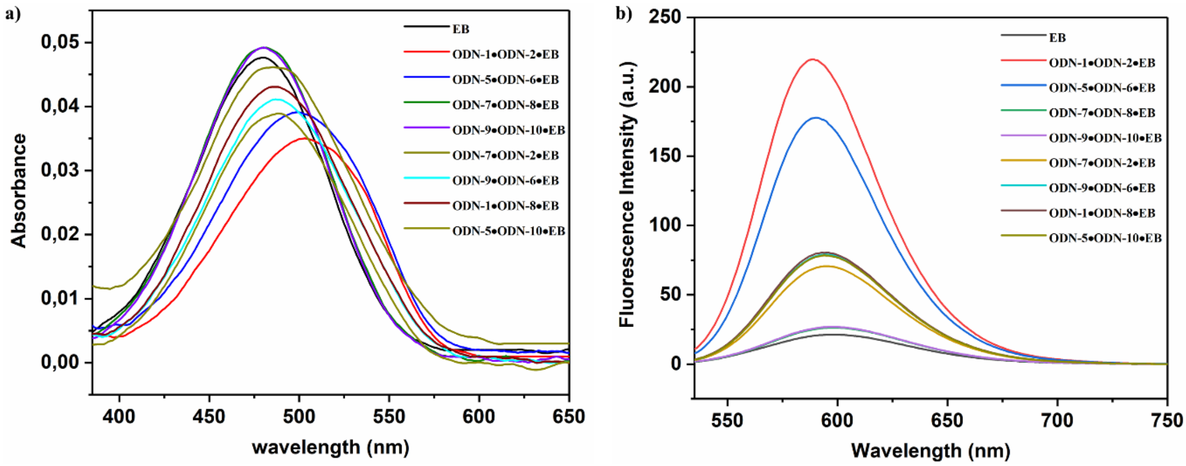

β-5’-d(TAG GTC AAT ACT) (ODN-**1**)
β-3’-d(ATC CAG TTA TGA) (ODN-**2**)

β-5’-d(TCA TAA CTG GAT) (ODN-**5**)
β-3’-d(AGT ATT GAC CTA) (ODN-**6**)

α-5’-d(TCA TAA CTG GAT) (ODN-**7**)
α-3’-d(ATC CAG TTA TGA) (ODN-**8**)

α-5’-d(TAG GTC AAT ACT) (ODN-**9**)
α-3’-d(ATC CAG TTA TGA) (ODN-**10**)

α-5’-d(TCA TAA CTG GAT) (ODN-**7**)
β-5’-d(AGT ATT GAC CTA) (ODN-**2**)

β-5’-d(TAG GTC AAT ACT) (ODN-**1**)
α-5’-d(ATC CAG TTA TGA) (ODN-**8**)

α-5’-d(TAG GTC AAT ACT) (ODN-**9**)
β-5’-d(ATC CAG TTA TGA) (ODN-**6**)

β-5’-d(TCA TAA CTG GAT) (ODN-**5**)
α-5’-d(AGT ATT GAC CTA) (ODN-**10**)

Figure S9. a) UV absorption spectra for free EB and in the presence of antiparallel and parallel duplexes. b) Fluorescence emission spectra for free EB and in the presence of antiparallel and parallel duplexes. The EB concentration was 8.5 µM and the duplex concentration was 5 µM. Measurements were performed in 100 mM NaCl, 10 mM $MgCl_2$, 10 mM Na-cacodylate, pH 7.0.

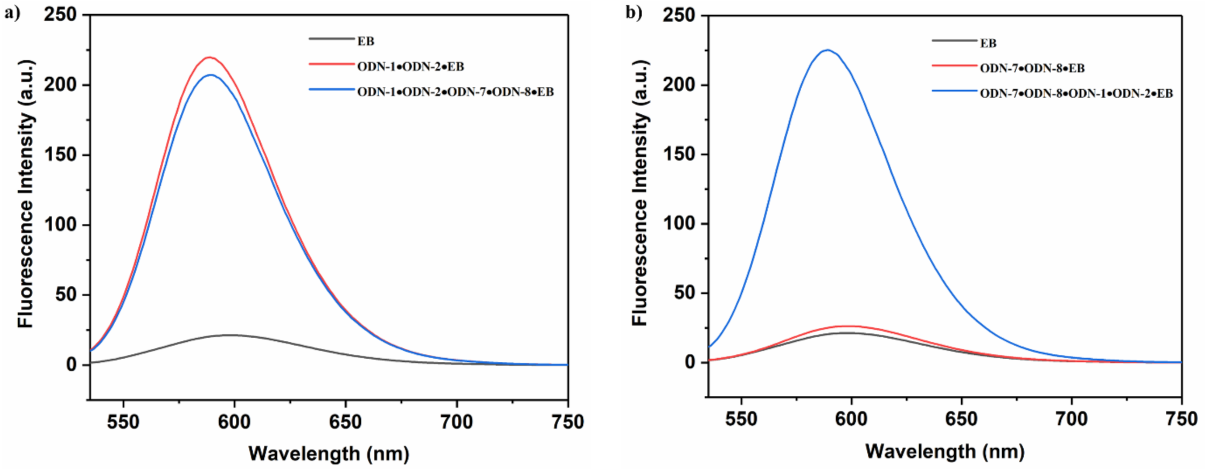

Figure S10. Fluorescence emission spectra for free EB and EB bound to antiparallel duplexes. a) ODN-**1**·ODN-**2** to ODN-**1**·ODN-**2**·ODN-**7**·ODN-**8**; b) ODN-**7**·ODN-**8** to ODN-**7**·ODN-**8**·ODN-**1**·ODN-**2**.

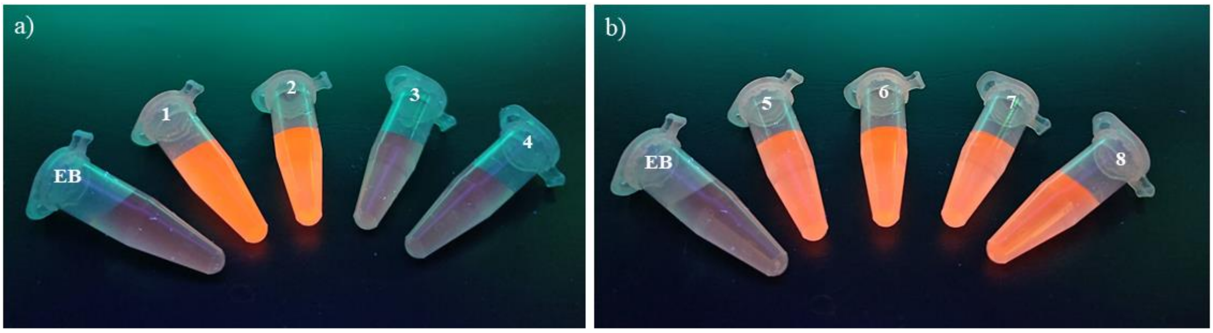

Figure S11. Fluorescence images for free EB and EB bound to antiparallel duplexes and parallel duplexes. 1) ODN-**1**·ODN-**2**; 2) ODN-**5**·ODN-**6**; 3) ODN-**7**·ODN-**8**; 4) ODN-**9**·ODN-**10**; 5) ODN-**1**·ODN-**2**·ODN-**7**·ODN-**8**; 6) ODN-**5**·ODN-**6**·ODN-**9**·ODN-**10**; 7) ODN-**7**·ODN-**8**·ODN-**1**·ODN-**2**; 8) ODN-**9**·ODN-**10**·ODN-**5**·ODN-**6**.

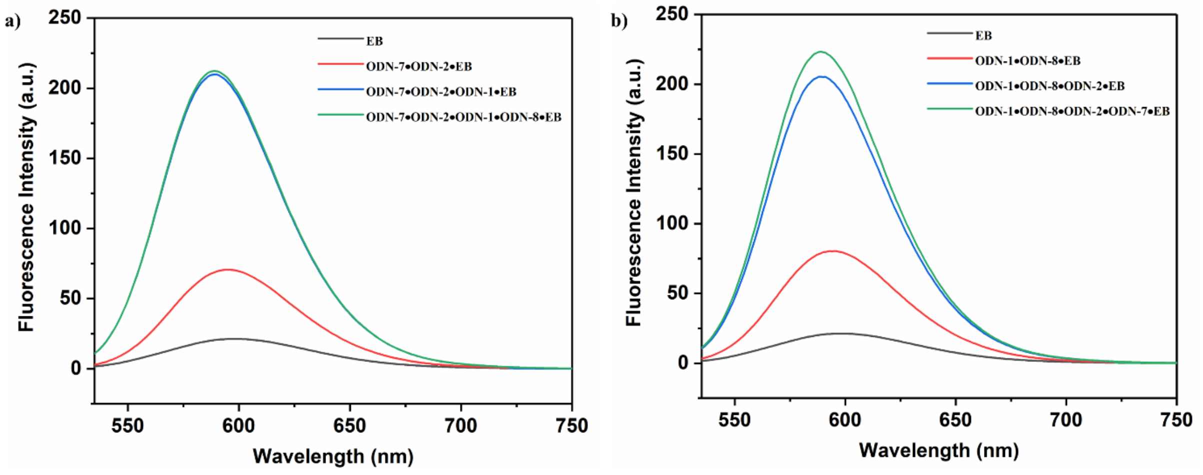

Figure S12. Fluorescence emission spectra for free EB and EB bound to parallel duplexes and parallel duplexes adding β and α oligonucleotides. a) ODN-**7**·ODN-**2** to ODN-**7**·ODN-**2**·ODN-**1** and ODN-**7**·ODN-**2**·ODN-**1**·ODN-**8**; b) ODN-**1**·ODN-**8** to ODN-**1**·ODN-**8**·ODN-**2** and ODN-**1**·ODN-**8**·ODN-**2**·ODN-**7**.

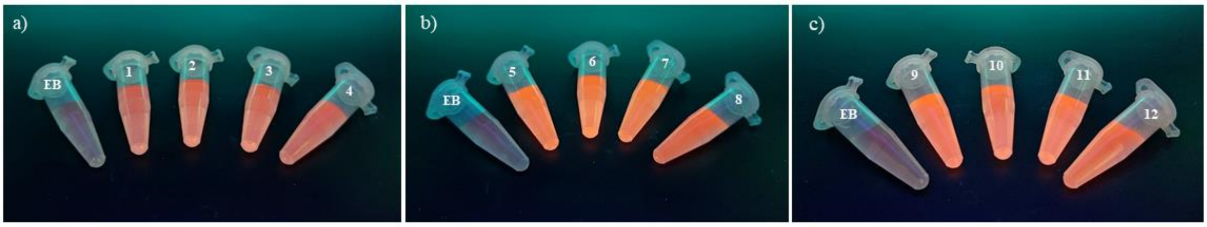

Figure S13. Fluorescence images for free EB and EB bound to parallel duplexes and parallel duplexes after adding β-D and α-D invader strands. 1) ODN-**7**·ODN-**2**; 2) ODN-**9**·ODN-**6**; 3) ODN-**1**·ODN-**8**; 4) ODN-**5**·ODN-**10**; 5) ODN-**7**·ODN-**2**·ODN-**1**; 6) ODN-**9**·ODN-**6**·ODN-**5**; 7) ODN-**1**·ODN-**8**·ODN-**2**; 8) ODN-**5**·ODN-**10**·ODN-**6**; 9) ODN-**7**·ODN-**2**·ODN-**1**·ODN-**8**; 10) ODN-**9**·ODN-**6**·ODN-**5**·ODN-**10**; 11) ODN-**1**·ODN-**8**·ODN-**2**·ODN-**7**; 12) ODN-**5**·ODN-**10**·ODN-**6**·ODN-**9**.

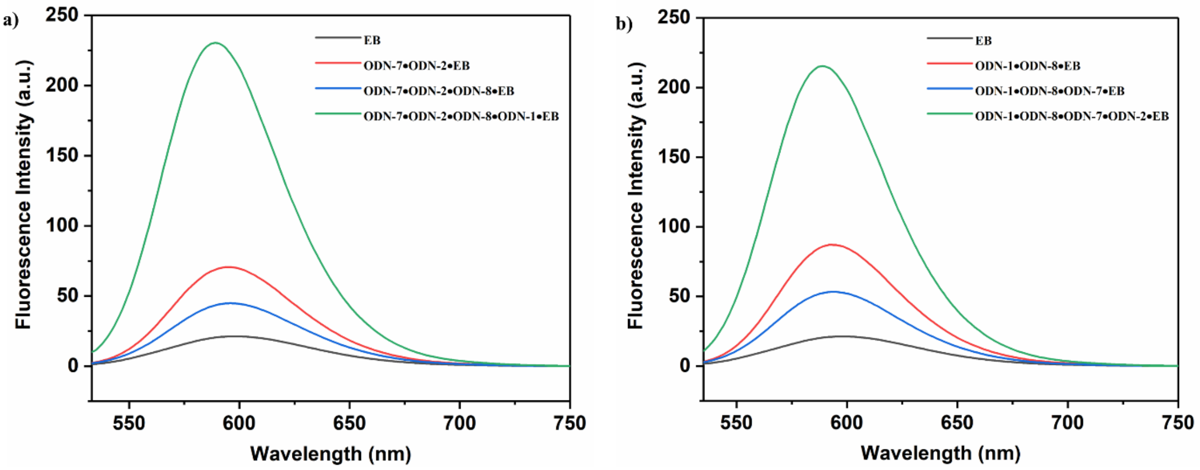

Figure S14. Fluorescence emission spectra for free EB and EB bound to parallel duplexes and parallel duplexes after adding α-D and β-D invader strands. a) ODN-**7**·ODN-**2** to ODN-**7**·ODN-**2**·ODN-**8** and ODN-**7**·ODN-**2**·ODN-**8**·ODN-**1**; b) ODN-**1**·ODN-**8** to ODN-**1**·ODN-**8**·ODN-**7** and ODN-**1**·ODN-**8**·ODN-**7**·ODN-**2**.

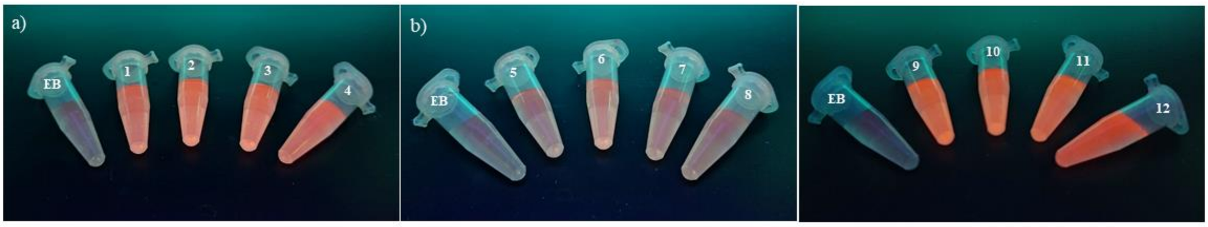

Figure S15. Fluorescence images for free EB and EB bound to parallel duplexes and parallel duplexes after adding α-D and β-D invader strands. 1) ODN-**7**·ODN-**2**; 2) ODN-**9**·ODN-**6**; 3) ODN-**1**·ODN-**8**; 4) ODN-**5**·ODN-**10**; 5) ODN-**7**·ODN-**2**·ODN-**8**; 6) ODN-**9**·ODN-**6**·ODN-**10**; 7) ODN-**1**·ODN-**8**·ODN-**7**; 8) ODN-**5**·ODN-**10**·ODN-**9**; 9) ODN-**7**·ODN-**2**·ODN-**8**·ODN-**1**; 10) ODN-**9**·ODN-**6**·ODN-**10**·ODN-**5**; 11) ODN-**1**·ODN-**8**·ODN-**7**·ODN-**2**; 12) ODN-**5**·ODN-**10**·ODN-**9**·ODN-**6**.

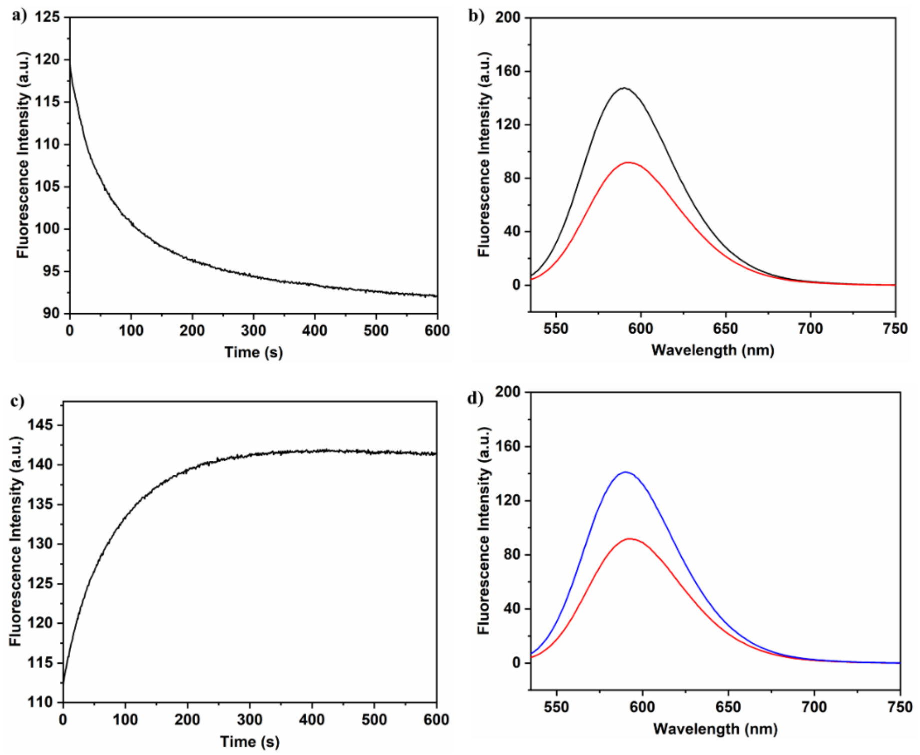

System 1

β-5’-d(TCATAACTGGAT) + α-5’-d(AGTATT GACCTA) → β-5’-d(TCATAACTGGAT)
β-3’-d(AGTATTGAC……) (Invader) α-5’-d(AGTATTGACCTA)

System 2

β-5’-d(TCATAACTGGAT) + α-5’-d(TAGGTCAATACT) → α-5’-d(AGTATTGACCTA)
α-5’-d(AGTATTGACCTA) (Invader) α-3’-d(TCATAACTGGAT)

Figure S16. Reaction progress of displacement reactions followed by the fluorescence change of ethidium bromide (extrapolated curve). a) System 1; c) system 2. Steady-state fluorescence emission spectra of the starting duplex plus EB and the final duplex plus EB. b) System 1; d) system 2. All measurements were performed at 260 nm with 5 µM single-strand concentration and 8.5 µM ethidium bromide in 100 mM NaCl, 10 mM $MgCl_2$, and 10 mM Na-cacodylate (pH 7.0)

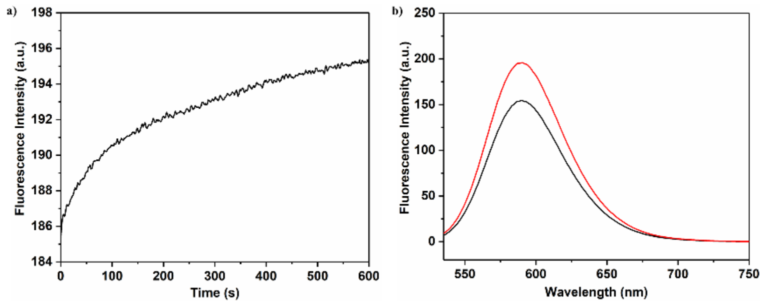

System 3

β-5’-d(TCATAACTGGAT) + β-5’-d(ATCCAGTTATGA) → β-5’-d(TCATAACTGGAT) + β-3’-d(AGTATTGAC……)
β-3’-d(AGTATTGAC……) (Invader) β-3’-d(AGTATTGACCTA)

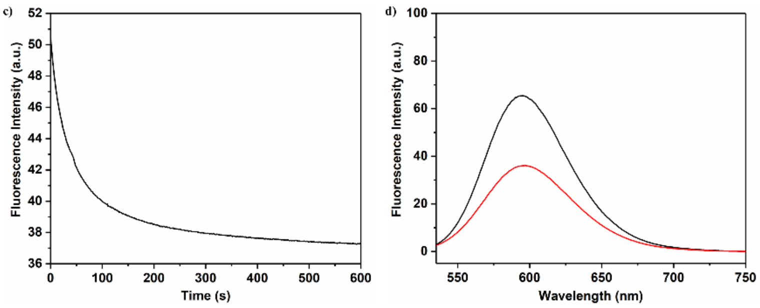

System 4

α-5’-d(TCATAACTGGAT) + α-5’-d(ATCCAGTTATGA) → α-5’-d(TCATAACTGGAT) + β-5’-d(AGTATTGACCTA)
β-5’-d(AGTATTGACCTA) (Invader) α-3’-d(AGTATTGACCTA)

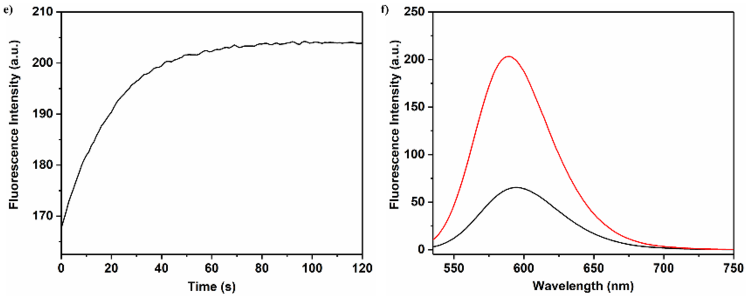

System 5

α-5’-d(TCATAACTGGAT) + β-5’-d(TAGGTCAATACT) → β-5’-d(TAGGTCAATACT) + β-5’-d(AGTATTGACCTA)
β-5’-d(AGTATTGACCTA) (Invader) β-5’-d(ATCCAGTTATGA)

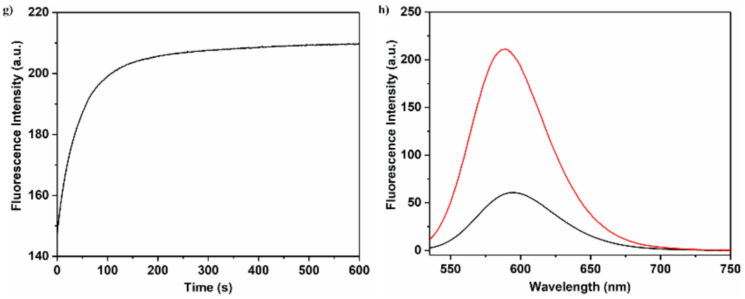

System 6

α-5’-d(TCATAACTGGAT) + β-5’-d(TAGGTCAATACT) → β-5’-d(TAGGTCAATACT) + α-5’-d(TCATAACTGGAT)
β-5’-d(AGTATTGACCTA) α-5’-d(ATCCAGTTATGA) β-3’-d(ATCCAGTTATGA) α-3’-d(AGTATTGACCTA)
(Invader)

Figure S17. Reaction progress (extrapolated curve) of the displacement reaction according to a) system 3; c) system 4; e) system 5; g) system 6. Steady-state fluorescence emission spectra of the starting duplex plus EB and the final duplex plus EB b) system 3; d) system 4; f) system 5; h) system 6.
